# CM363, a novel naphthoquinone derivative which acts as multikinase modulator and overcomes imatinib resistance in chronic myelogenous leukemia

**DOI:** 10.18632/oncotarget.11425

**Published:** 2016-08-19

**Authors:** Borja Guerra, Patricia Martín-Rodríguez, Juan Carlos Díaz-Chico, Grant McNaughton-Smith, Sandra Jiménez-Alonso, Idaira Hueso-Falcón, Juan Carlos Montero, Raquel Blanco, Javier León, Germán Rodríguez-González, Ana Estévez-Braun, Atanasio Pandiella, Bonifacio Nicolás Díaz-Chico, Leandro Fernández-Pérez

**Affiliations:** ^1^ Instituto de Investigaciones Biomédicas y Sanitarias BioPharm Laboratory, Universidad de Las Palmas de Gran Canaria, Epaña; ^2^ Unidad de Apoyo a la Docencia en Enfermería-Fuerteventura, Universidad de Las Palmas de Gran Canaria, España; ^3^ Instituto Canario de Investigación sobre el Cáncer, España; ^4^ Centro Atlántico del Medicamento, España; ^5^ Centro de Investigación del Cáncer, CSIC, Universidad de Salamanca, España; ^6^ Instituto de Biomedicina y Biotecnología de Cantabria, CSIC-Universidad de Cantabria, España; ^7^ Departamento de Química Orgánica, Instituto de Bio-Orgánica Antonio González, Universidad de la Laguna, España

**Keywords:** leukemia, naphthoquinone, Bcrl-Abl-Stat5, imatinib resistance

## Abstract

Human Chronic Myelogenous Leukemia (CML) is a hematological stem cell disorder which is associated with activation of Bcr-Abl-Stat5 oncogenic pathway. Direct Bcr-Abl inhibitors are initially successful for the treatment of CML but over time many patients develop drug resistance. In the present study, the effects of CM363, a novel naphthoquinone (NPQ) derivative, were evaluated on human CML-derived K562 cells. CM363 revealed an effective cell growth inhibition (IC50 = 0.7 ± 0.5 μM) by inducing cancer cells to undergo cell cycle arrest and apoptosis. CM363 caused a dose- and time-dependent reduction of cells in G0/G1 and G2/M phases. This cell cycle arrest was associated with increased levels of cyclin E, pChk1 and pChk2 whereas CM363 downregulated cyclin B, cyclin D3, p27, pRB, Wee1, and BUBR1. CM363 increased the double-strand DNA break marker γH2AX. CM363 caused a time-dependent increase of annexin V-positive cells, DNA fragmentation and increased number of apoptotic nuclei. CM363 triggered the mitochondrial apoptotic pathway as reflected by a release of cytochrome *C* from mitochondria and induction of the cleavage of caspase-3 and -9, and PARP. CM363 showed multikinase modulatory effects through an early increased JNK phosphorylation followed by inhibition of pY-Bcrl-Abl and pY-Stat5. CM363 worked synergistically with imatinib to inhibit cell viability and maintained its activity in imatinib-resistant cells. Finally, CM363 (10 mg/Kg) suppressed the growth of K562 xenograft tumors in athymic mice. In summary, CM363 is a novel multikinase modulator that offers advantages to circumvent imanitib resistance and might be therapeutically effective in Bcrl-Abl-Stat5 related malignancies.

## INTRODUCTION

Chronic Myelogenous Leukemia (CML) is a hematological stem cell disorder characterized by excessive proliferation of cells of the myelogenous lineage [1]. The hallmark of CML is the Philadelphia chromosome, which arises from reciprocal translocation between chromosomes 9 and 22. The molecular consequence of this translocation is the replacement of the first exon of c-Abl with sequences from the Bcr gene resulting in a Bcr-Abl fusion gene whose protein product generates a constitutively activated tyrosine kinase. Found in 95% of patients with CML, Bcr-Abl is also present in approximately 5–10% of adults with acute leukemia for whom there is no evidence of antecedent CML. Bcr-Abl is considered necessary, but may not be sufficient, to cause malignant transformation in CML. Bcr-Abl activates intracellular signal transduction pathways that promote proliferation and genetic instability while suppressing apoptosis and weakening cellular adhesion [1]. Biochemical signaling pathways known to be activated by Bcr-Abl include the PI3K/AKT/mTOR pathway, CRK oncogene-like protein/focal adhesion kinase, the RAS/RAF/MEK/ERK pathway, c-Jun NH2-terminal kinase/stress-activated protein kinase (JNK/SAPK), and the Signal Transducer and Activator of Transcription (Stat)-5 pathway [1–5]. Particularly, activation of Stat5 by Bcr/Abl leads to increased expression of genes driving cell cycle progression, promoting survival, and oncogenesis signaling pathways downstream of Bcr-Abl kinase [6]. In contrast, deletion of Stat5 in Bcr-Abl^+^ cells induces apoptosis, even in Bcr-Abl tyrosine kinase inhibitors (TKI)-resistant cells. Furthermore, Stat5 is constitutively active in many forms of hematologic cancers [5], and, aside Bcr-Abl, it is a signaling hallmark of CML and its activity is associated with poor prognosis [7].

The tyrosine kinase inhibitor (TKI) imatinib mesylate (IM), an ATP-competitive selective inhibitor of Bcr-Abl, is the standard first-line therapy for all CML patients [8]. Particularly, 80% of newly diagnosed patients with chronic-phase CML have shown a complete cytogenetic response to treatment with IM over a median follow-up of 54 months. However, although the initial response rates are high, IM fails in up to 40% of patients because of disease resistance or unacceptable side effects which call for alternative therapies to treat CML patients. Both preclinical and clinical studies suggest that the induction of the apoptosis of Bcr-Abl^+^ leukemia cells by IM may be incomplete and patients can develop resistance to TKI [9]. The most frequently reported causes for TKI resistance are mutations in the kinase domain of Bcr-Abl. Other mechanisms include the increased expression of proteins such as Bcr-Abl or the drug transporter ABCB1, TP53 inactivation, elevated levels of granulocyte-macrophage-colony-stimulating factor (GM-CSF), or increased Stat5 activation [9]. To circumvent the resistance, more potent TKI have been approved (e.g., dasatinib and nilotinib). However, these compounds do not show therapeutic activities against all IM-resistant mutants of Bcr-Abl, and finally a long-term tolerability problem has emerged [8, 10]. Preclinical and clinical studies suggest that multikinase drugs (i.e., to target alternate Bcr-Abl kinase pathways) might produce better results than “selective” TKI because mitogenesis and drug resistance can occur, in part, via activation of alternate Bcr-Abl mitogenic signals [10, 11]. Therefore, the combination of TKI with multikinase inhibitors would be clinically relevant. Notably, the inhibition of phosphoStat5 (pStat5) constitutes a suitable target to abrogate CML cell growth and other types of leukemias, and, it is considered an attractive target to overcome resistance to clinically used Bcr-Abl kinase inhibitors [12–14].

The biological activities and structural properties of naphthoquinone (NPQ) (mainly 1,4-napthtoquinones and their derivatives) have led to consider them as privileged structures in medicinal chemistry. NPQ-based derivatives have exhibited a wide variety of biological activities which include, among others, anti-inflammatory, cytotoxic, and anticancer activities. Particularly, NPQ derivatives have been shown to suppress Stat signaling pathway in cancer cells [15, 16]. Recently, we have discovered the compound CM363 [4-(1,6,11-trioxo-2,3,4,6,11,12-hexahydro-1H-benzo[b]xanthen-12-yl)benzonitrile] [17], a synthetic NPQ-based derivative that was discovered by transcriptionally based assays and phenotypic cell based screening of a small molecules library. In this study, we report the anti-CML effects of CM363 *in vitro* and *in vivo*. CM363 was characterized as an inhibitor Bcr-Abl-Stat5 signaling pathway that induces cell cycle arrest and apoptosis in CML cells. These effects are enhanced when CM363 is combined with IM. Notably, CM363 is equally effective against IM-sensitive and IM–resistant cells. These findings provide new insights into molecular and cellular mechanisms of a novel multikinase modulator in CML and suggest a potential therapeutic application of this compound in Bcr-Abl- and Stat5-related malignancies.

## RESULTS

### CM363 inhibits viability of human leukemia cells

**Figure 1:**
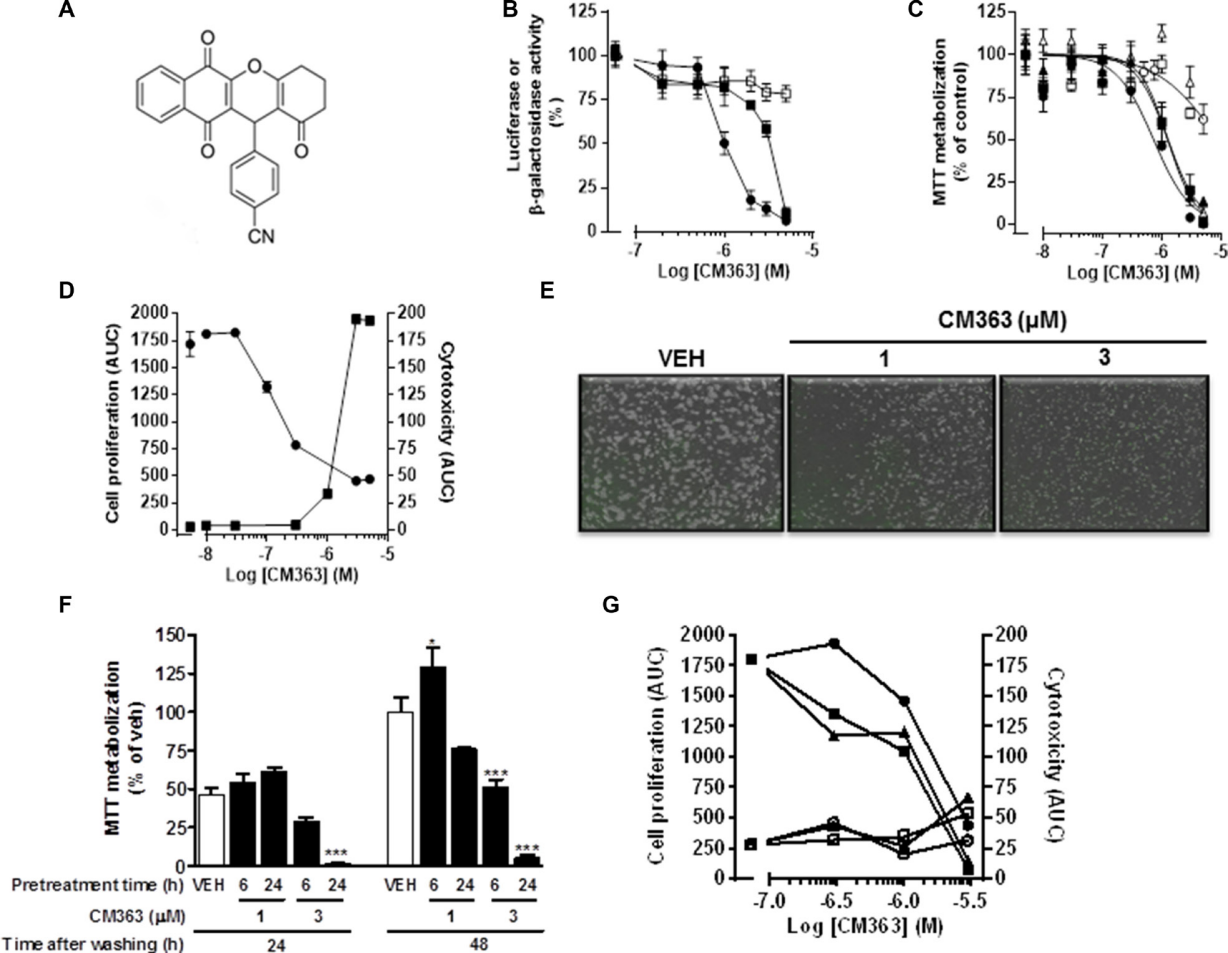
CM363 reduces viability and growth of human leukemia cells (**A**) Chemical structure of CM363; (**B**) Serum starved HEKGHR and HeLa/Stat3-luc cells were used to interrogate chemical library on Stat5 ∙ and Stat3 (■) response element driving expression of a luciferase reporter gene, respectively. The expression vector for β-galactosidase protein (□) was used to control transfection efficiency. Then, cells were pretreated with vehicle or CM363 for 1 h followed by GH (for Stat5) or IL6 (for Stat3) for 7 h. Luciferase activity was measured as described in Material and Methods. (**C**) Cells were cultured in the presence of the indicated concentrations of CM363 for 48 h, and thereafter cell viability of K562 ∙, HEL (■), HL60 (▲), Hela (Δ), MRC5 (□), and PMBC (○) cells were determined by the MTT assay; (**D**) K562 cells were cultured in the absence (vehicle) or presence of the indicated concentrations of CM363 over 4-day period. The effects of CM363 on K562 cell proliferation ∙ and cytotoxicity (■) were studied by using the Incucyte HD real-time system and data are represented as area under curve (AUC); (**E**) Representative photomicrographs of exponentially growing K562 cells in the absence (vehicle; VEH) or presence of CM363 for 48 h; (**F**) Exponentially growing K562 cells were pulsed-exposed to 1 or 3 μM CM363 for either 6 or 24 h. Then, K562 cells were washed and grown in the absence of CM363 for additional 24 or 48 h, and cell viability was studied by using MTT assay; (**G**) Exponentially growing K562 cells were pulsed-exposed to 0.3, 1 or 3 μM CM363 for either 2(∙,○), 6 (■,□) or 24 (▲,Δ) h. Then, K562 cells were washed and cell proliferation (black symbols) and cytotoxicity (white symbols) were studied in the absence of CM363 for additional 3 days by using Incucyte HD real-time system. Data are represented as area under curve (AUC). Figures are representative of 2–3 independent experiments each one performed in triplicate. ^**^*P < 0.001 *versus* vehicle-treated cells (VEH); *P < 0.05 *versus* vehicle-treated cells (VEH).

CM363 (Figure 1A) was discovered by high-throughput cell based phenotypic screening of a proprietary small molecule library [17]. First, cells transfected with a Stat-response element driving expression of a luciferase reporter gene were used to interrogate that chemical library. From this approach, CM363 was found to inhibit Stat5- or Stat3- (Figure 1B) dependent reporter genes. However, CM363 had negligible effects on transcriptional activity that regulates constitutive expression of β-galactosidase or luciferase (data not shown) protein (Figure 1B), suggesting that CM363 does not inhibit transcription factors broadly. Second, viability of exponentially growing cells, representative of hematological and solid tumors, was studied in the presence of CM363 for 48 h by using the MTT assay. CM363 was able to inhibit cell viability of all human leukemia cell lines tested (i.e., K562, MOLM.13, MV4.11, HEL, and HL60) with high efficacy (Table 1). Thus, IC50s for K562 (IC50 = 0.7 ± 0.5 μM), HEL (IC50 = 1.3 ± 0.3 μM), and HL60 (IC50 = 1.3 ± 0.6 μM) cells (Figure 1C) were lower than that observed for some non-hematological (i.e., HeLa = 4.8 ± 0.4 μM) and non-malignant (i.e., MRC-5 = 3.5 ± 0.01 μM and PBMC cells > 5 μM) cells (Table 1 and Figure 1C). *In vivo* Live-Cell Imaging of K562 cells corroborated that CM363 (Figure 1D) caused a cytostatic effect on cell growth at concentrations lower than 1 μM (IC50_AUC_ = 0.6 ± 0.3 μM) and induced a cytotoxic effect at higher concentrations (EC50_AUC_ = 1.1 ± 0.4 μM). As expected [18], IM caused a cytostatic effect on K562 cells growth (IC50_AUC_ = 0.2 ± 0.1 μM) (data not shown). Time-lapse movies and photomicrograph of each well confirmed the effects of CM363 on K562 cell proliferation (Figure 1E). Finally, viability and proliferation of K562 cells were examined after cells were pulsed-exposed to 1–3 μM CM363 for either 6–24 h, followed by CM363 removal from medium, and then grown in the absence of CM363 for additional 1–2 days. Exposure of K562 cells to 3 μM CM363 for 6 h followed by 48 h of cells cultured in CM363-free culture medium, caused a significant decrease of K562 cell viability (Figure 1F). Furthermore, when the effects of transient exposure to CM363 were analyzed by using the Live-Cell Imaging System (Figure 1G), we observed that 2 h of transient exposure to CM363 (IC50_AUC_ = 1.9 ± 0.5 μM) was enough to cause a cytostatic effect on K562 cells for additional 72 h. Taken together, these results suggest that CML cells are acutely sensitive to CM363 and that they cannot overcome the inhibitory effects on cell growth caused by a short-transient exposure to this novel NPQ derivative.

**Table 1:** Effects of CM363 on blood and non-blood cancer cells

Cell Line	IC50 (μM)
	Mean ± SD
Raw	0.1 ± 0.2
HEK	0.6 ± 0.2
K562	0.7 ± 0.5
MOLM.13	0.7 ± 1.1
NCTC	1.1 ± 0.7
MV4.11	1.2 ± 1.1
HEL	1.3 ± 0.3
HL60	1.3 ± 0.6
T47D	1.3 ± 1.2
PC3	1.5 ± 1.0
MG63	1.6 ± 0.9
MCF7	1.7 ± 1.1
SKBR3	2.6 ± 0.8
HCT-15	3.3 ± nd
L1210	3.4 ± 0.4
U-2 OS	4.3 ± 1.2
HeLa	4.8 ± 0.4
MRC.5	3.5 ± 0.0
PMBC	> 5

Cells were treated with vehicle (0.05% DMSO) or CM363 (0.01 to 10 μM) for 48 h. Cell viability was assessed by MTT assay as described in Material and Methods (nd: not determined).

### CM363 blocks cell cycle progression in human chronic myelogenous leukemia cells

**Figure 2:**
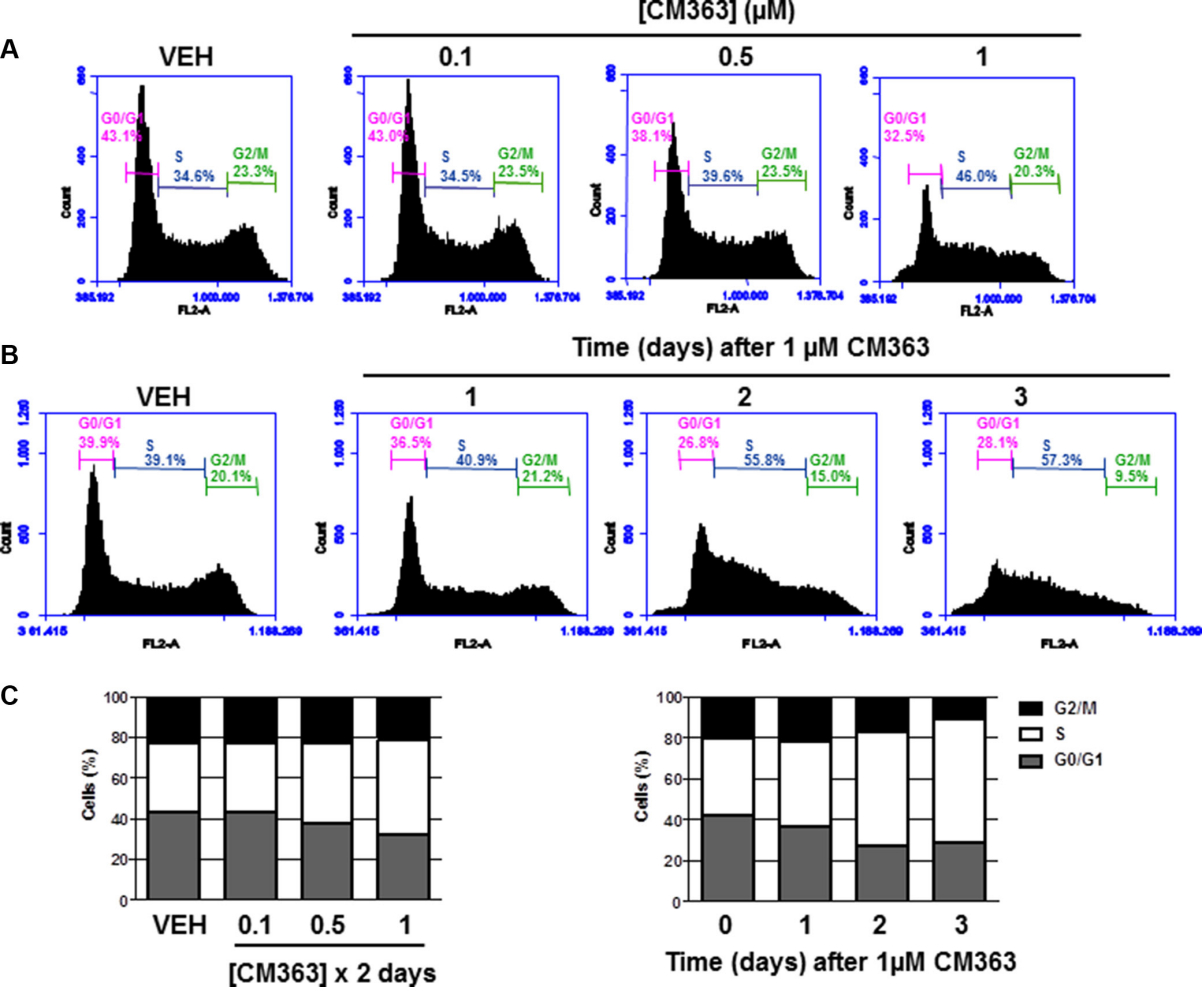
CM363 blocks cell cycle progression in human chronic myelogenous leukemia cells K562 cultures were treated with different doses of CM363 (**A**) or with 1 μM CM363 for 1–3 days (**B**). Subpopulation of cells (**C**) were quantified as percentage of cells in the sub-regions G0, M o S by fluorescence flow cytometry as described in Material and Methods. Figures are representative of two independent experiments each one performed in triplicate.

**Figure 3:**
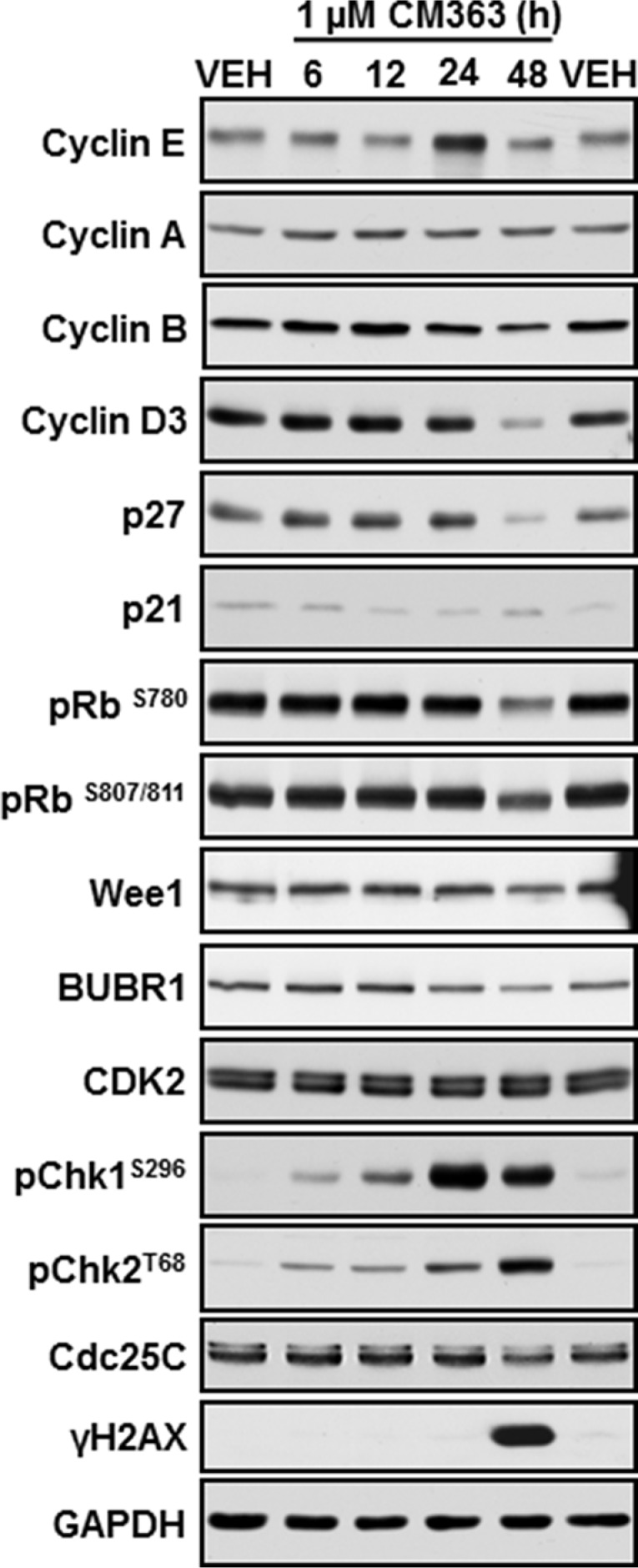
CM363 modulates proteins involved in cell cycle regulation K562 cells cultures were exposed for 6, 12, 24, 48 hours to CM363 (1 μM). Then, whole cell extracts were obtained and proteins involved in cell cycle regulation were analyzed by immunoblotting. Figures are representative of two independent experiments.

To assess whether the decrease of the K562 cell growth induced by CM363 was the result of cell cycle blockade, an increase in cytotoxicity, or both, K562 cells were treated with CM363 (0.1–1 μM) for different times and cell cycle profiles and apoptotic induction were analyzed. CM363 caused an increase in S phase and a reduction in G0/G1 and G2/M phases (Figure 2A–2C). To further investigate the mechanism of action of CM363, we analyzed the changes induced by this compound on proteins involved in cell cycle regulation [19, 20]. Blockage of cell cycle was associated with increased levels of cyclin E and increased phosphorylations of Checkpoint kinase (Chk)-1 and Chk2 (Figure 3). Notably, the expression level of phosphatase Cdc25C, which plays a critical role in the G2/M checkpoint [19], was reduced by CM363 (Figure 3). CM363 also reduced amounts of cyclin B, cyclin D3, p27, Wee1, BUBR1 as well as phosphorylation of retinoblastoma protein (Rb) whereas CDK2 levels remained unaffected (Figure 3). Importantly, CM363 increased the double-strand DNA break marker γH2AX which indicates that K562 cells cannot overcome cell cycle arrest and that they are destined for apoptosis (Figure 3).

### CM363 induces apoptosis in human chronic myelogenous leukemia cells

**Figure 4:**
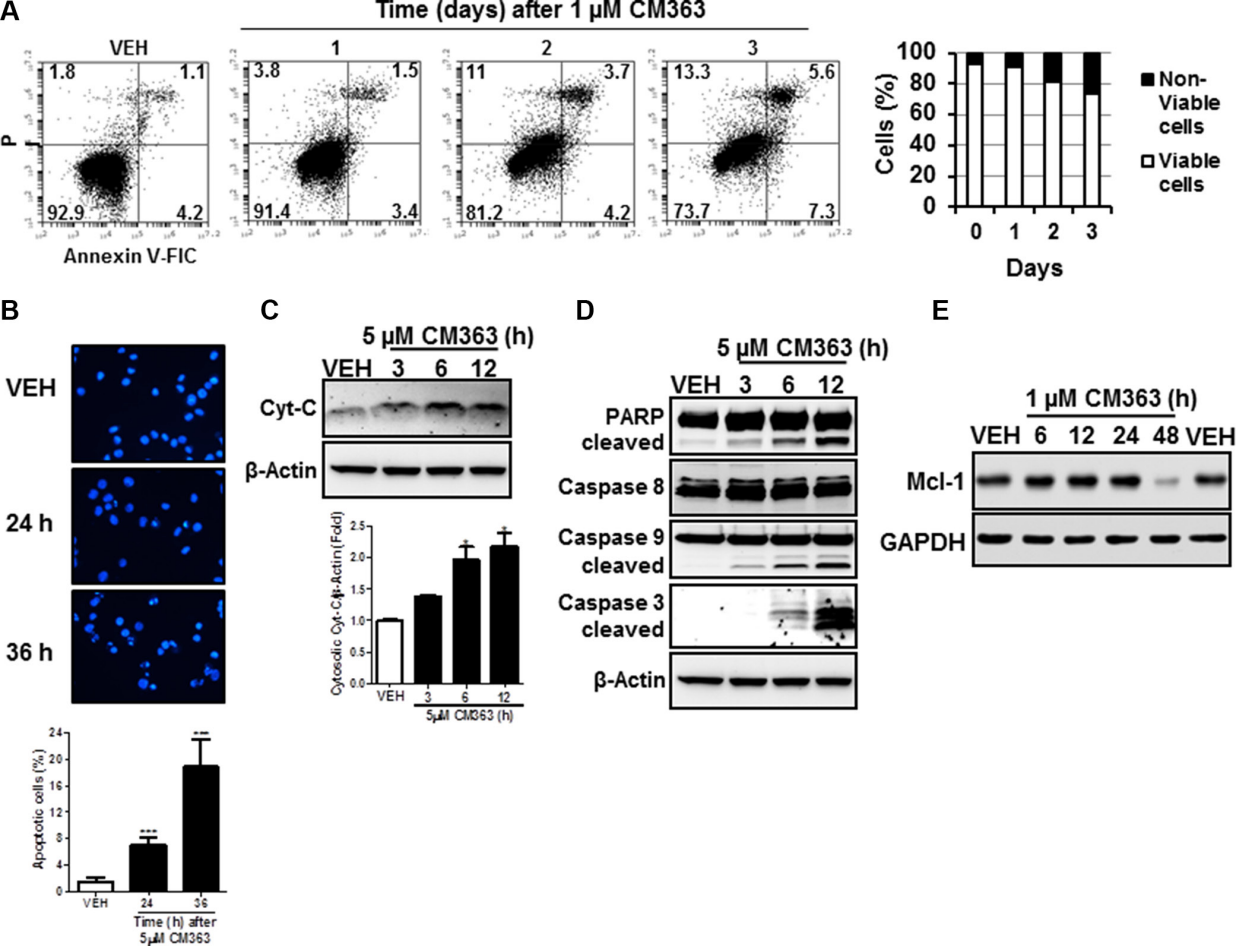
CM363 induces apoptosis in K562 cells (**A**) K562 were exposed for 1, 2, 3 days to CM363 (1 μM) to determine translocation of phosphatidylserine to the cell surface using the annexin V-FITC; (**B**) Photomicrographs of representative fields of K562 cells stained with bisbenzimide trihydrochloride to evaluate nuclear chromatin condensation after treatment for 24 and 36 h with CM363 (5 μM); K562 cultures were exposed for different times to CM363 (5 μM) and (**C**) cytosolic (cytochrome C) or (**D**) whole cell (PARP and pro-caspases 3, -8, and -9) extracts were analyzed by immunoblotting. Densitometric results from immunosignal values of cytosolic citochrome c relative to β-Actin are shown. β-Actin was used as a loading control. (**E**) Time-dependent effects of CM363 on Mcl-1 were studied by immunobloting. Figures are representative of two-three independent experiments. ^**^*P < 0.001 *versus* vehicle-treated cells (VEH); *P < 0.05 *versus* vehicle-treated cells (VEH).

In addition to cell cycle arrest, CM363 reduced viability of K562 cells was associated with a time-dependent increase of annexin V-positive cells (Figure 4A) and increased number of apoptotic nuclei (Figure 4B). CM363 also caused a time dependent release of cytochrome *C* from mitochondria (Figure 4C) and induction of the cleavage of caspase-3, -9, and poly(ADP-ribose) polymerase (PARP) (Figure 4D), which suggests that CM363 triggered the mitochondrial apoptotic pathway [21]. Finally, we observed that CM363 reduced the expression level of myeloid cell leukemia 1 (Mcl-1), an anti-apoptotic member of the Bcl-2 family of proteins [22, 23].

### CM363 inhibits Bcr-Abl-Stat5 and induces JNK signaling pathways in human chronic myelogenous leukemia cells

**Figure 5:**
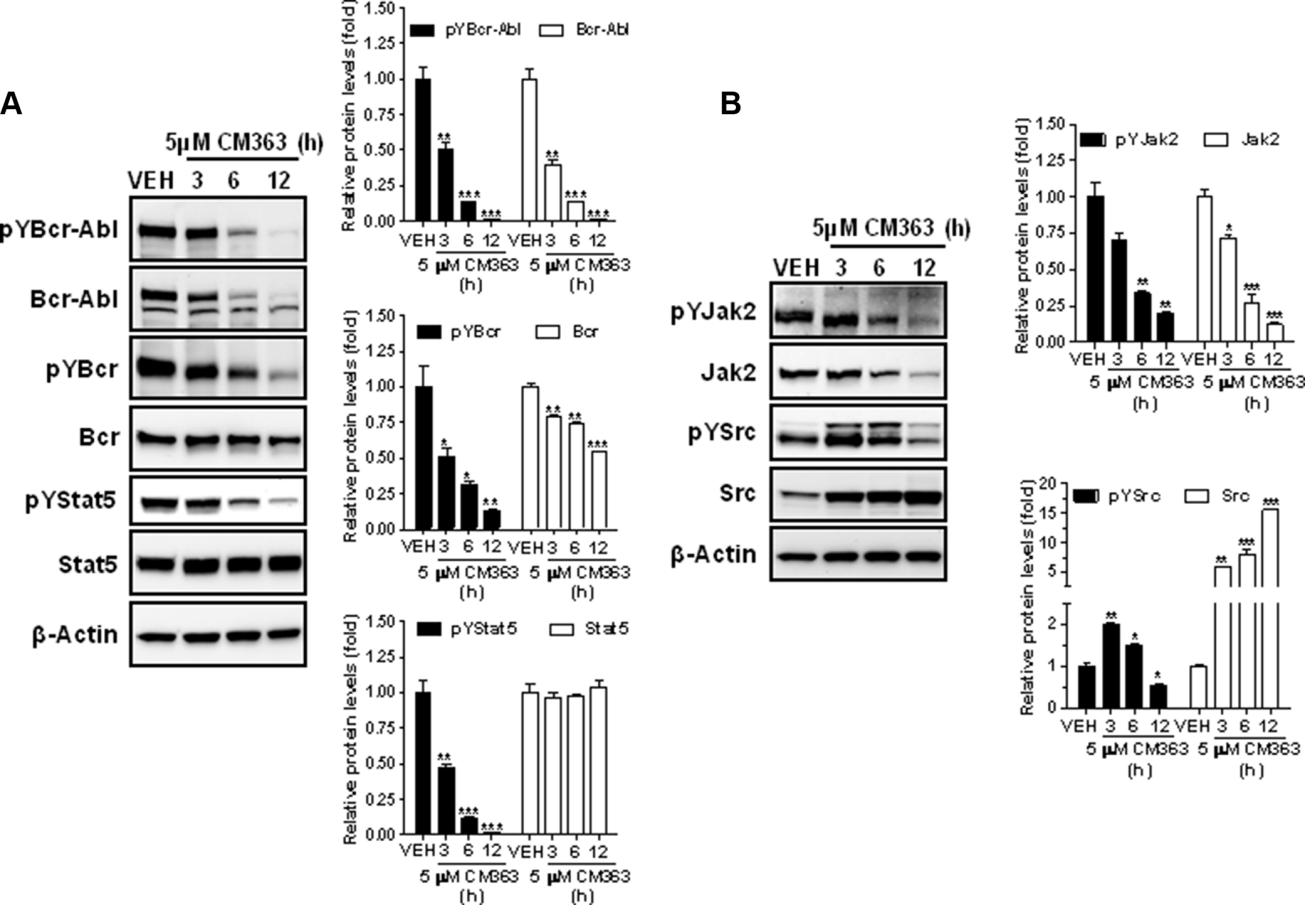
CM363 inhibits constitutive Bcrl-Abl-Stat5 signaling pathway (**A**) K562 cultures were incubated with vehicle (VEH) or CM363 (5 μM) for indicated times. Then, immunoblotting analyzes were performed to detect the phosphorylated (pY) and total levels of Bcr-Abl, Bcr (**upper and middle panel, respectively**) and Stat5 (**lower panel**). (**B**) K562 cultures were incubated with vehicle or CM363 (5 μM) for indicated times. Then, immunoblotting analyzes were performed to detect the phosphorylated (pY) and total forms levels of Jak2 and Src proteins. Densitometric cuantification from immunosignal values of phospho and total proteins relative to β-Actin (VEH: fold 1) are shown. Figures are representative of two independent experiments each one performed in duplicate. ^**^*P < 0.001 *versus* vehicle-treated cells (VEH). ^*^P < 0.01 *versus* vehicle-treated cells (VEH); *P < 0.05 *versus* vehicle-treated cells (VEH).

**Figure 6:**
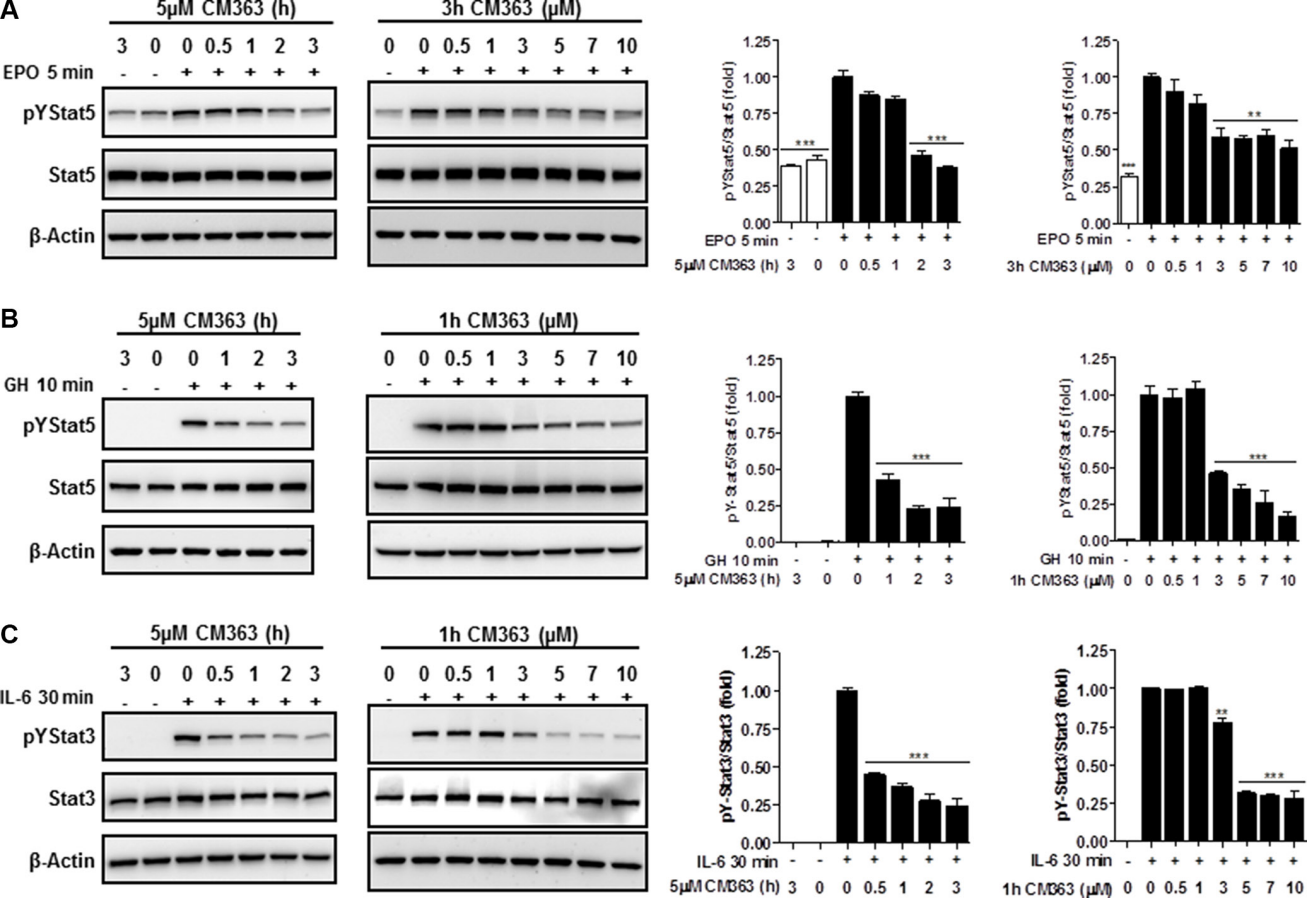
CM363 inhibits cytokine-induced tyrosine phosphorylations of Stat5 and Stat3 (**A**) Serum-deprived HEL cells were pre-incubated with CM363 (5 μM) for indicated times (**left panel**) or treated with different doses of CM363 for 3 h (**right panel**). Then, HEL cells were treated with EPO (10 units/ml) for 5 min followed by immunoblotting analyses of phosphorylated (pY) and total forms of Stat5. Densitometric analyses of pYStat5 relative to total Stat5 levels are shown. (**B**) Serum-deprived T47D cells were pre-incubated with CM363 (5 μM) for indicated times (**left panel**) or treated with different doses of CM363 for 1 h (**right panel**). Then, T47D were treated with GH (50 nM) for 10 min followed by immunoblotting analyses of phosphorylated and total Stat5 levels. Densitometric analyses of pYStat5 relative to total Stat5 levels are shown. (**C**) Serum-deprived T47D cells were pre-incubated with CM363 (5 μM) for indicated times (**left panel**) or treated with different doses of CM363 for 1 h (**right panel**). Then, T47D were treated with IL-6 (25 ng/ml) for 30 min followed by immunoblotting analyses of phosphorylated (pY) and total Stat3 levels. Densitometric analyses of pYStat3 relative to total Stat3 levels are shown. Figures are representative of two independent experiments each one performed in duplicate. ^**^*P < 0.001 *versus* EPO (A), GH (B) or IL-6 (C) stimulated cells; ^*^P < 0.01 *versus* EPO (A) or IL-6 (C) stimulated cells.

**Figure 7:**
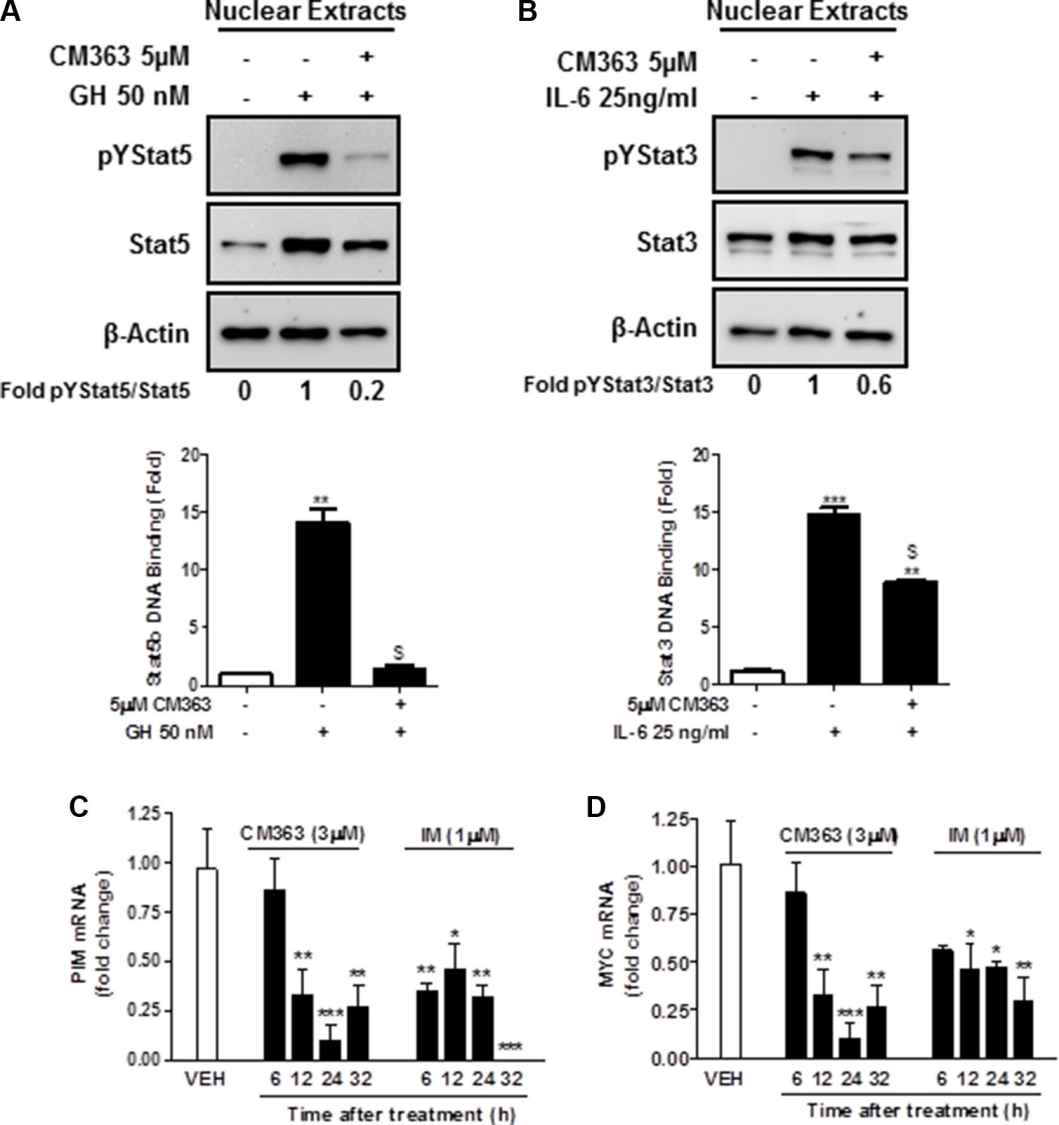
CM363 inhibits cytokine-induced DNA-binding activities of Stat5 and Stat3 (**A**) Serum-deprived T47D cells were pre-incubated with CM363 (5 μM) for 1 h followed by GH (50 nM) for 10 min or (**B**) by IL-6 (25 ng/ml) for 30 min. Then, nuclear protein extracts were assayed by immunoblotting to detect the phosphorylated and total levels of Stat5 (**A**; **upper panel**) and Stat3 (**B**; **upper panel**), respectively. Nuclear proteins from CM363 and GH/IL-6-treated T47D cells were analyzed to measure the binding of Stat5b (**A**; **lower panel**) or Stat3 (**B**; **lower panel**) to DNA by using ELISA as described in Materials and Methods. β-Actin was used as a loading control. K562 cells were incubated with vehicle (VEH), CM363 (3 μM) or IM (1 μM) for indicated times. Then, mRNA levels of (**C**) PIM and (**D**) c-Myc were analyzed. Figures are representative of two independent experiments each one performed in triplicate.*P < 0.05 *versus* vehicle-treated cells; ^*^P < 0.01 *versus* vehicle-treated cells; ^$^*P* < 0.01 *versus* GH (A) or IL-6 (B) stimulated cells; ^**^*P < 0.001 *versus* vehicle-treated cells.

Several cell survival signaling pathways in CML cells have been shown to be modulated by Bcr-Abl, including Jak/Stat, JNK, and PI3K/AKT/mTOR signaling pathways [3]. Therefore, we first analyzed the effects of CM363 on Bcrl-Abl-Stat5 signaling pathway. Constitutive activation of pTyr^694^-Stat5 (pYStat5) and pTyr^177^-Bcrl-Abl (pYBcr-Abl), which are critical for K562 cell survival, were significantly inhibited after exposure to 5 μM CM363 for 6 h (Figure 5A). The inhibitory effect of CM363 on Bcrl-Abl was mainly due to reduced Bcr-Abl protein content. Interestingly, CM363-induced Bcr-Abl and Stat5 inhibition was associated with reduced pTyr^1007/1008^-Jak2 (pYJak2) and increased pTyr^416^-Src (pYSrc), mainly caused by declined and increased Jak2 and Src total protein content, respectively (Figure 5B). To further support the hypothesis that CM363 was able to inhibit constitutive pYStat5 in leukemia cells, we assessed the effects of CM363 on HEL, a human erythroleukemia cell line harboring constitutive activation of Jak2/Stat5 signaling pathway [24]. Accordingly, CM363 also inhibited pYStat5 in a time- and dose-dependent manner in HEL cells (data not shown). To support the hypotheses that CM363 was also able to inhibit cytokine-induced Stat5/3 activities, we first observed that when HEL cells were serum starved, EPO caused an increase of pYStat5 within 5 min, whereas CM363 inhibited this activity in a time- and dose-dependent manner (Figure 6A). Next, we used T47D cells, a human breast adenocarcinoma cell line which lacks constitutively active Stat but is biologically responsive to GH and IL6 which are activators of Stat5 and Stat3 signaling pathway, respectively [25, 26]. T47D cells where treated with GH or IL6 at different times and then examined for pYStat5 (Figure 6B) or pTyr^705^-Stat3 (pYStat3) (Figure 6C), respectively. GH induced pYStat5 within 10 minutes whereas IL-6 induced pYStat3 within 30 min. Exposure of cells to CM363 for 1 h was sufficient to suppress GH-induced pYStat5 (Figure 6B) as well as IL-6-induced pYStat3 (Figure 6C). In agreement with inhibition of pYStat, CM363 suppressed GH- and IL-6-induced binding to DNA of Stat5 (Figure 7A) and Stat3 (Figure 7B), respectively. In addition, exposition of primary mouse macrophages to 3 μM CM363 for 0.5 h caused complete inhibition of GM-CSF-induced (10 min) pYStat5 (data not shown). Finally, functional consequences of CM363 on K562 cells were shown by decreased mRNA levels of PIM (Figure 7C) and c-Myc (Figure 7D), two target genes of Bcrl-Abl-Stat5 signaling pathway [1].

**Figure 8:**
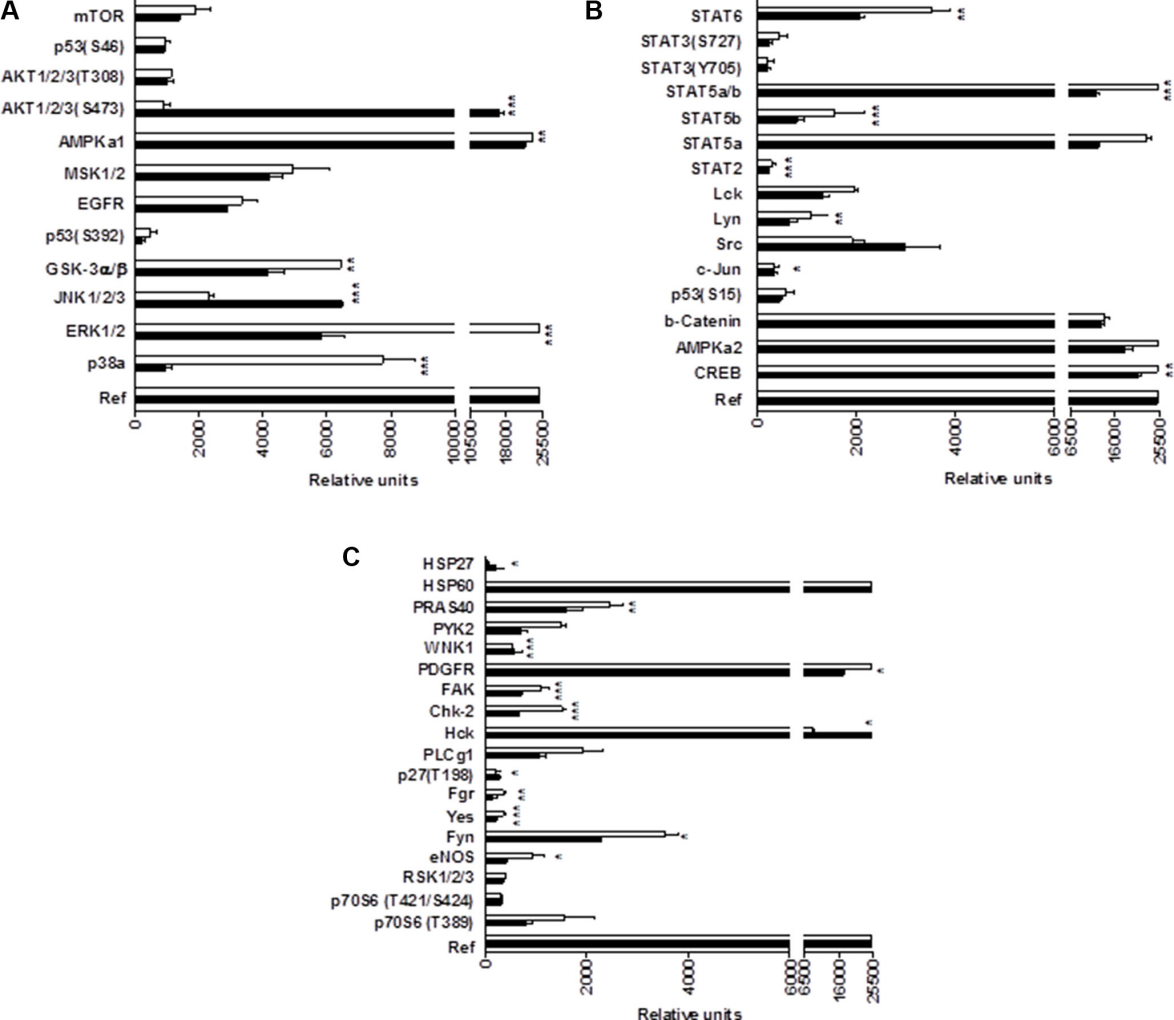
Phosphoprotein analysis array in CM363-treated K562 cells (**A–C**) K562 cultures were incubated with vehicle (VEH, □) or CM363 (5 μM, ■) for 6 h. Then, cell whole extracts were analyzed by using the Human Phospho-Kinase Array kit as described in Material and Methods. ^*^P < 0.01 *versus* vehicle-treated cells (VEH); ^**^*P < 0.001 *versus* vehicle-treated cells (VEH).

**Figure 9:**
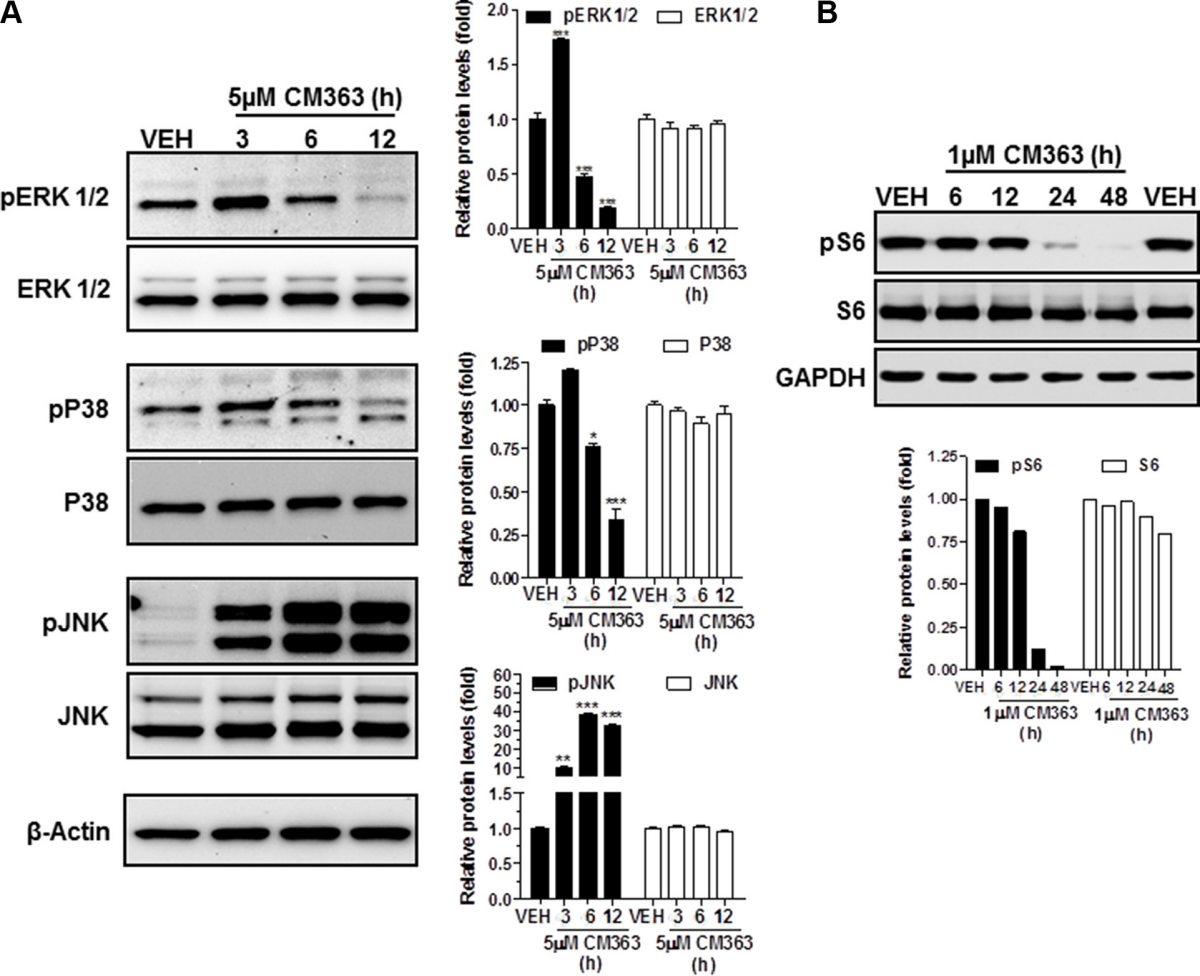
CM363 modulates survival signaling pathways K562 cultures were incubated with vehicle (VEH) or CM363 (5 μM) for indicated times. Then, immunoblotting analyzes were performed to detect the phosphorylated and total levels of (**A**) ERK1/2, P38-MAPK, JNK and (**B**) pS6. β-Actin was used as a loading control (VEH: fold 1). Figures are representative of two independent experiments each one performed in duplicate.*P < 0.05 *versus* vehicle-treated cells (VEH, □). ^*^P < 0.01 *versus* vehicle-treated cells (VEH, □); ^**^*P < 0.001 *versus* vehicle-treated cells (VEH, □).

In summary, these results indicate that CM363 can inhibit both constitutive as well as cytokine-induced activation of Stat signaling pathway. Next, we analyzed the effects of CM363 on several RTKs and relevant signaling mediators in K562 cells. This analysis was performed after exposure of K562 cells to 5 μM CM363 for 6 h, by using an antibody-based Human Phospho-Kinase Array kit. The analyses revealed that of the proteins evaluated, only some of them were modified after exposition of K562 cells to CM363 (Figure 8A–8C). The most inhibited proteins involved member of MAPK (p38; ERK1/2), Stat (Stat5b) and cell cycle (Chk-2) signaling pathways; whereas CM363 increased phosphorylation of JNK (JNK1/2/3) and AKT [(Ser^473^-AKT1/2/3)] proteins. In accordance with Human Phospho-Kinase Array analyses, CM363 decreased the levels of pERK1/2 and p38MAPK under the level of phosphorylation in vehicle-treated cells (Figure 9A). Notably, the activation (phosphorylation) of JNK, which appears crucial for Bcr-Abl–induced cellular proliferation and transformation [27], was evident as early as 3 h after CM363 treatment and persisted for 12 h (Figure 9A). The expression levels of total forms of ERK1/2, p38MAPK or JNK were no altered after CM363 treatment. Finally, the modulatory effects of CM363 on components of the PI3K/AKT/mTOR pathway were also studied. Particularly, CM363 reduced S6 phosphorylation levels (pS6) [28] (Figure 9B), which suggests inhibition of protein synthesis in K562 cells [29].

### Coadministration of CM363/IM interacts synergistically to inhibit cell viability in CML, circunvent resistance of CML to IM

**Figure 10:**
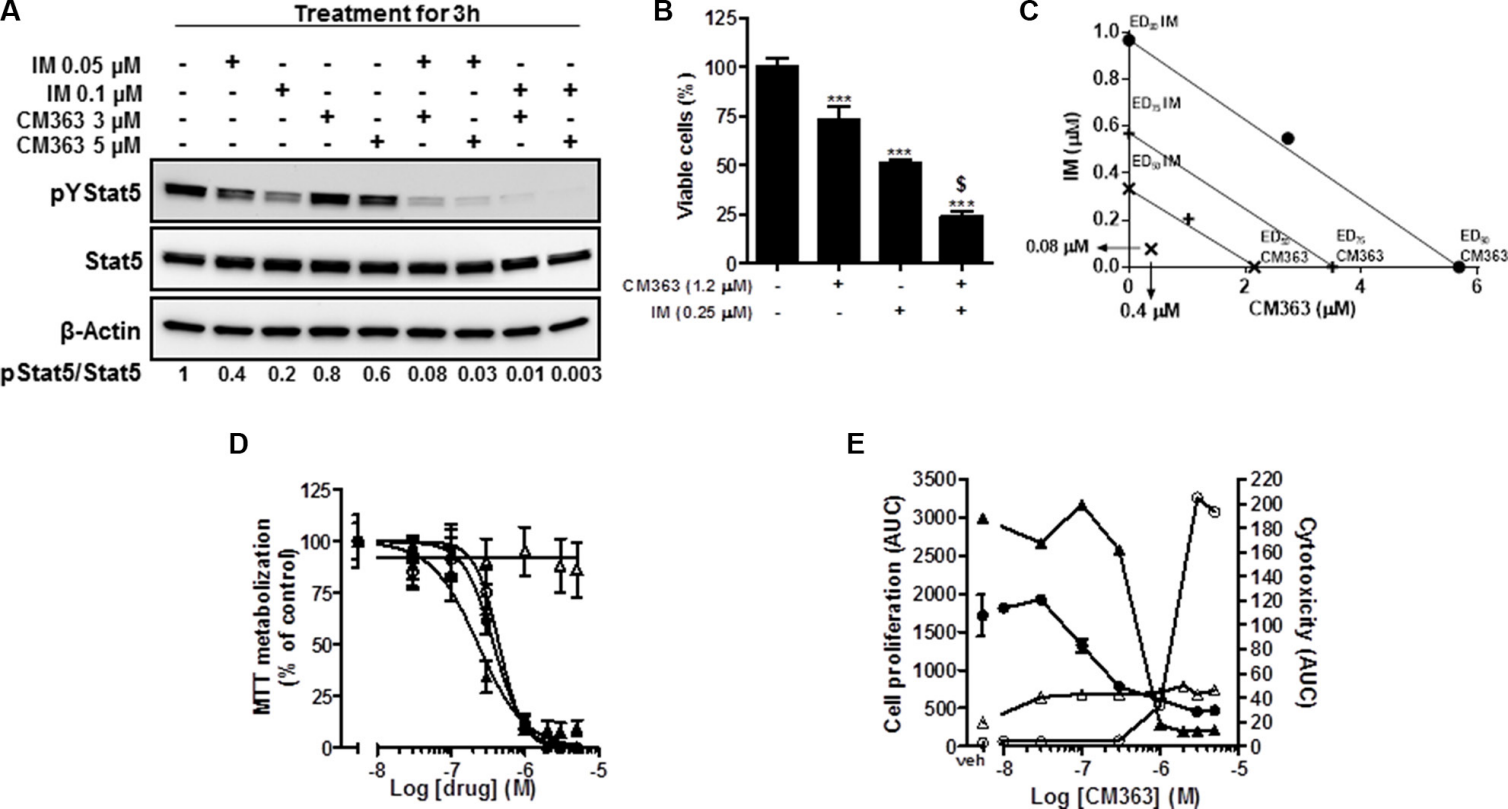
Coadministration of CM363/IM interacts synergistically to inhibit pTyr^694^-Stat5, cell viability in CML and to circunvent resistance of CML to IM (**A**) K562 cultures were incubated with CM363 (3 or 5 μM), IM (0.05 or 0.1 μM) or both for 3 h, after which total protein extracts were assayed by immunoblotting using specific antibodies directed against the phosphorylated and total forms of Stat5. β-Actin was used as a loading control. (**B**) K562 cells were cultured in the presence of CM363 (1.2 μM), IM (0.25 μM) or both for 48 h, and K562 cells survival were analyzed by the MTT assay. (**C**) K562 cultures were treated with CM363 (8 doses; from 0 to 5 μM), IM (8 doses; from 0 to 1 μM) or both in combination (constant ratio combination design (5 (CM363):1 (IM)) for 48 h, after which viable cell number was measured by MTT assay as described in Materials and Methods. Isobologram analysis was performed based on change in viable cell number for 8 different doses of CM363 and IM using the software Calcusyn^®^ as described in Materials and Methods. Data are representative of three independent experiments. (**D**) The IM sensitive K562 cells (▲,■) and IM-resistant K562-R (Δ,○) cells were treated with increasing concentrations of CM363(∙,○) or IM (▲, Δ) for 72 h. Then, cell viability was measured by MTT assay as described in Materials and Methods. (**E**) IM sensitive K562 cells(∙,○) or IM resistance cells (▲, Δ) were cultured in the absence (vehicle) or presence of the indicated concentrations of CM363 over 4-day period. The effects of CM363 on cell proliferation(∙,▲) and cytotoxicity (○, Δ) were studied by using the Incucyte HD real-time system. Data are represented as area under curve (AUC). Figure is representative of two-three independent experiments each one performed in triplicate. ^**^*P < 0.001 versus vehicle treated cells (VEH); *^$^P < 0.001* versus CM363 or IM treated cells.

To investigate whether a CM363/IM combination might provide enhanced activity, we firstly studied the effects of this combination on pYStat5. Exposition of K562 cells for 3 h to 3 μM CM363 and 0.05 μM IM, individually, exerted 20% and 40% inhibition on pYStat5. However, when these two drugs were combined virtually eliminated pYStat5 (Figure 10A). Secondly, we investigated a potential enhanced action of a CM363/IM combination on inhibiting cell survival. When CM363 and IM were combined we observed a greater inhibition of cell survival compared to either drug used alone (Figure 10B). In another set of experiments, we utilized a constant ratio combination design [5(CM363):1(IM)] to determine whether these two drugs may act synergistically on inhibiting K562 cells survival. Isobologram and Chou-Talalay analysis of the combination index [30] showed that CM363 and IM acted synergistically in inducing antiproliferative effects (combination index (CI) for ED_50_ = 0.4 ± 0.2 and CI for ED_75_ = 0.6 ± 0.3) (Figure 10C). To assess the broadness of the CM363 inhibitor, we determined its activity against IM-resistant CML-derived cell lines. The IM resistant K562-R [31] and KBTI [32] cells and the IM sensitive K562 cells counterpart were treated with increasing concentrations of CM363 or IM for 24–96 h. Notably, IC50 of IM needed for inhibit viable cells was almost 25 times higher in the K562-R cells than in the IM sensitive K562 cells counterpart (Figure 10D). However, both K562 as well as K562-R cells showed equivalent sensitivity to CM363. Inhibition was detected for concentration of CM363 as low as 0.3 μM, and the IC50 values were close to 0.6 μM for both clones. CM363 was also active against IM-resistance KBTI human cells [32] (data not shown). Interesting, KBTI cells showed a partial resistance to CM363, suggesting that CM363 has also some inhibitory activity against the Bcr-Abl kinase expressed by CML cells. *In vivo* Live-Cell Imaging of K562 cells corroborated that CM363 caused a cytostatic effect on IM sensitive (IC50_AUC_ = 0.6 ± 0.3 μM) as well as IM resistant (IC50_AUC_ = 0.5 ± 0.1 μM) K562 cells at concentrations lower than 1 μM (Figure 10E). Surprisingly, higher concentration of CM363 caused a cytotoxic effect on IM sensitive K562 cells whereas it was cytostatic on IM resistance K562-R cells (Figure 10E).

### CM363 inhibits growth of human CML xenografts

**Figure 11:**
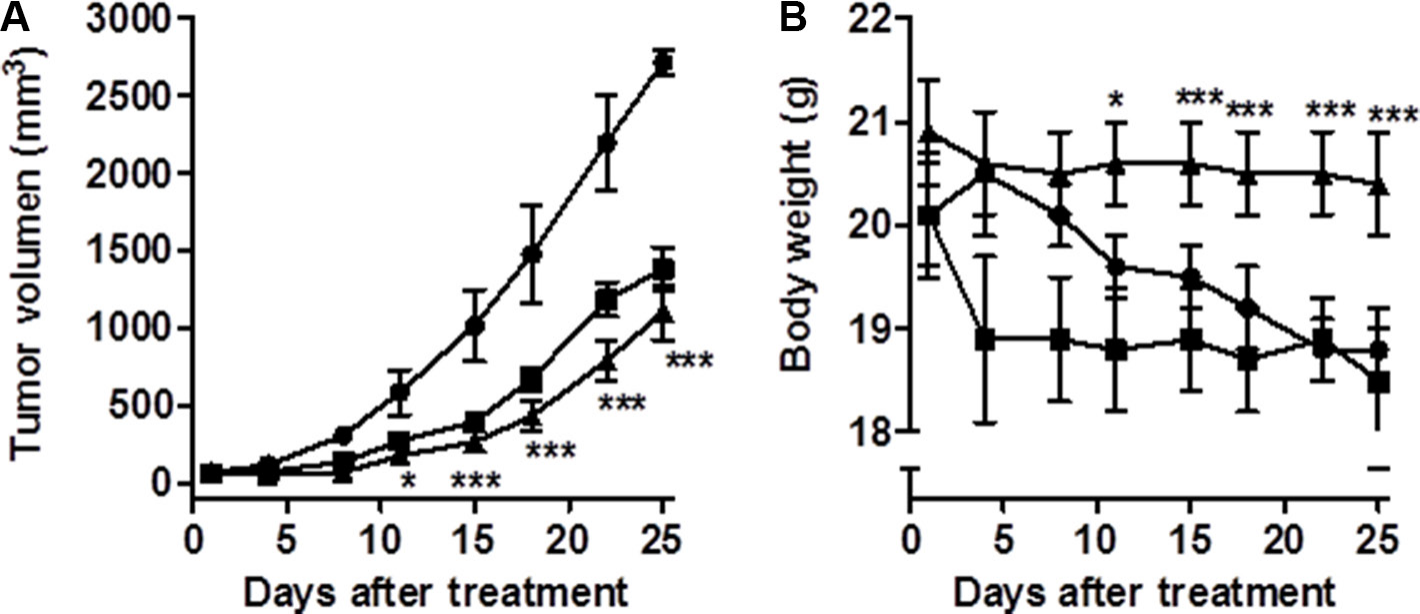
CM363 inhibits growth of human CML xenografts *in vivo* Human K562 cells were inoculated subcutaneously into flanks of mice as described in Materials and Methods. Then, mice were divided into three groups of six mice each: vehicle-treated(∙), CM363 (10 mg/kg/daily; ip) (▲) and IM (40 mg/kg/daily; oral) (■). Volumes of xenograft tumors (**A**) and body weights (**B**) were measured twice a week. One-way ANOVA followed by Tukey’s multiple comparison tests were used to compare vehicle (control) with drug-treated group. *P < 0.05 versus vehicle-treated mice; ^**^*P < 0.001 versus vehicle-treated mice.

Finally, we also determined whether CM363 administration could suppress the growth of K562 tumor xenografts in NOD-SCID mice [33]. Volume of the tumors established in mice in the control group (vehicle) increased in a consistent manner throughout the 25 days study period (Figure 11A). As expected the administration of the positive control compound IM (40 mg/kg, orally) produced a pronounced and statistically significant reduction (*P* = 0.039 day 4 and *P* = 0.008 day 25, compared to control group) in tumor growth (Figure 11A). Administration of CM363 (10 mg/kg, i.p.) also produced a pronounced, and statistically significant reduction (*P* = 0.004 day 4 and *P* = 0.007 day 25 compared to control group) in tumor growth (Figure 11A). Interestingly, no significant body weight reduction was associated with CM363 treatment (Figure 11B).

## DISCUSSION

The NPQ-based derivatives are considered as privileged chemical structures to develop antitumor agents [15, 16]. In fact, some quinone-antitumor agents have been used to treat solid tumors (e.g., doxorubicin) or acute lymphoblastic and myeloblastic leukemias (e.g., daunorubicin). Particularly, NPQ derivatives (e.g., shikonin, plumbagin, LQB-118) produce CML cell death with μM, low μM or sub-μM IC_50_ values [34–36]. However, the potential effects of these NPQ derivatives on normal or non-malignant human cells were not investigated, so we cannot rule out the possibility that they may cause certain degree of toxicity in non-cancer cells. In the present work, we investigated, *in vitro* and *in vivo*, the potential antileukemic properties of a new NPQ-based derivative named CM363. The structural features shared by CM363 with other well-known kinase inhibitors (e.g., CYT387, WP1066, LY5, tofacitinib, imatinib) are cyano groups and aromatic rings [37–40]. CM363 effectively inhibits the growth of human hematological cancer cells with low μM (HL60 and HEL cells) or sub- μM (K562 cells) IC_50_, and with a higher efficacy than on other hematological or non-hematological cancer cells. In contrast, human PBMC and non-malignant lung fibroblast MRC-5 were less sensitive to inhibition of cell viability by CM363. Interestingly, CM363 was able to induce long term inhibitory effects on K562 cell growth after they were transiently exposed to CM363. These data suggest an anti-cancer selectivity for leukemia cells and that CML cells are acutely sensitive to NPQ-based derivative CM363.

The molecular hallmark of CML is the presence of the constitutively activated tyrosine kinase Bcr-Abl, which is the causative factor in the pathophysiology of CML [1]. Bcr-Abl/Jak2/Stat5 activity in CML leads to increased expression of genes driving promoting survival, cell cycle progression, and oncogenic signaling pathways [5, 12–14]. However, both preclinical and clinical studies suggest that cure of CML patients by direct TKI may be incomplete, because of disease resistance to therapy and unacceptable side effects [8]. Interestingly, Jak2/Stat5 signaling pathway is activated in IM-sensitive as well as IM-resistance Bcr-Abl^+^ cells [10]. In contrast, deletion of Jak2 or Stat5 in IM-sensitive and IM-resistant Bcr-Abl^+^ cells resulted in reduced colony formation and apoptosis [41]; therefore, Jak2/Stat5 inhibitors may be useful for CML therapy and the combination of TKI with CM363 might offer advantages to circumvent resistance to direct Bcr-Abl inhibitors. NPQ-based derivatives have been shown to suppress Stat signaling pathway in cancer cells [16, 34–36]. However, the potential effects of these compounds on Bcr-Abl/Jak2/Stat5 signaling pathway in CML have not been previously investigated. In the present study, we demonstrated that CM363 showed antitumoral efficacy in IM-sensitive as well as IM-resistant cells and that the combinatory effect of IM with CM363 was more effective to reduce viability and phosphorylation of Bcr-Abl/Stat5. Moreover, we also observed that CM363 reduced Jak2 phosphorylation in K562 cells. Previous studies have demonstrated that several NPQ-based derivatives can cause apoptosis in CML cells through induction of intrinsic mitochondria-dependent pathway [34, 36]. Similarly, we have demonstrated that exposure of the human CML cell line K562 to CM363 led to the induction of apoptosis as indicated by an increased annexin V binding as well as release of cytochrome c from the mitochondria into the cytoplasm, activation of caspases 3 and 9, and PARP cleavage. In contrast, CM363 did not activated caspase 8, which acts as initiator caspase in the extrinsic pathway of apoptosis. Previous studies have reported that Bcr-Abl inhibits apoptosis in CML cells through the regulation (expression and/or activation) of members of the Bcl-2 family of apoptosis modulators [22, 42, 43]. Particularly relevant in leukemia are the proapoptotic protein Bax, the antiapoptotic protein Bcl-xL, which prevents cytoplasmatic release of cytocrome c and, the antiapoptotic protein Mcl-1, which is able to bind to caspases in order to inhibit apoptosis signaling. Interestingly, it has been shown that Bcr-Abl/Stat5 inhibition induces apoptosis in CML cells by supressing Stat5-dependent transcriptional regulation of Bcl-2 family members such as Mcl-1 [22] and Bcl-xL [43]. In the present study, we showed that CM363 inhibited Bcr-Abl/Stat5 signaling pathway which was associated with decreased expression of Mcl-1 and increased expression of Bax and increased cytochrome realease from mitochondia. Therefore, one of the potential mechanisms that may explain the CM363 activation of intrinsic apoptotic pathway [44] in K562 cells is the dual inhibition of Bcr-Abl and Jak2 to whose activities Stat5 phosphorylation is linked. Bcr-Abl signaling is also linked to activation of the RAS/RAF/MEK/ERK, JNK, and the PI3K/AKT/mTOR pathways [1, 2, 4, 5]. The balance between the ERK1/2 cascade and p38MAPK and JNK pathways can determine whether the cells live or die. ERK1/2 in most cases exerts cytoprotective effects, whereas the JNK and p38 have generally been associated with pro-apoptotic actions [45]. However, p38MAPK signaling has also been shown to enhance survival, cell growth and differentiation. Therefore, the role of p38MAPK in apoptosis is dependent on cell types and stimuli [46]. Previous studies have reported that ERK1/2 inhibition causes apoptosis in K562 cells [47]. In the present work, we observed that Bcr-Abl inhibition caused by CM363 resulted in a downregulation of ERK1/2 phosphorylation which suggest that this mechanism may contribute to CM363-mediated apoptosis in K562 cells. Recent studies have shown that JNK activation is required for stress-induced release of mitochondrial cytochrome c and for apoptosis mediated by intrinsic signaling pathway [45]. Moreover, phosphorylation of prosurvival Bcl-2 by JNK disrupts the binding motif of Bcl-2 and makes it unable to antagonize Bax [48]. Interestingly, shikonin, a NPQ-based derivative, has been shown to induce the intrinsic apoptosis pathway in K562 cells through JNK activation [34]. In the current study we show that CM363 inhibits Bcr-Abl/Stat5 and induces JNK phosphorylation, so we cannot rule out the possibility that both mechanisms are contributing simultaneously to CM363-induced apoptosis. CM363 reduced cell viability was also associated with modulation of PI3K/AKT/mTOR pathway [3]. We observed that CM363 reduced S^424^S6, a ribosomal protein that is phosphorylated in a Bcr-Abl- and PI3K/mTOR-dependent manner in Bcr-Abl+ cell lines [28]. Furthermore, CM363 increased S^473^AKT, a phosphorylation that has been linked to inhibition of protein synthesis [29]. Therefore, these findings also support the hypothesis that the mechanism of CM363 action in K562 cells was linked, directly or indirectly, to inhibition of protein synthesis.

The mechanism of action of CM363 on K562 cells also involved arrest of cell cycle with augmented S phase and reduced G0/G1 and G2/M phases. These actions were associated with effects of CM363 on expression levels and/or phosphorylation of several proteins involved in cell cycle progression [20, 21, 49]. The onset of mitosis in the eukaryotic cell cycle requires the coordinated activation of multiple M phase-inducing protein kinases in a process in which the activation of cyclin-dependent kinase (CDK)-1/cyclin B complex plays a central role [19, 50]. Activation of this complex results from dephosphorylation of CDK1 by the protein phosphatase Cdc25. Interestingly, CM363 reduced the expression level of cyclin B and Cdc25. In addition, CM363 increased phosphorylation of Chk1 and Chk2, two checkpoint kinases that inactivate Cdc25 by phosphorylation or activates its degradation [19, 50]. Therefore, a reduced expression of Cdc25 together with an inactivation of Cdc25C by phosphorylation, most likely contribute to inhibition of mitotic entry by CM363. Moreover, many of the most potent CDC25 inhibitors are, similarly to CM363, quinone-containing compounds [50]. However, if a quinone-related mechanism is linked to the inhibitory effects of CM363 on cell cycle deserves further research. CM363-induced cell cycle arrest was also associated with decreased expression levels of p27/KIP and Wee1, as expected. The former two act as inhibitors of G1 and G2 CDKs, while the latter phosphorylates CDK1, inhibiting entry in mitosis. Both p21 and p27 have been shown to be required for the assembly of cyclin-CDK complexes. Therefore, in addition to their known action as CDK inhibitors, a certain level of these proteins is also required for the correct functioning of cyclin-CDK complexes essential for progression through the cell cycle [19, 50]. As mentioned above, CM363 reduces Mcl-1 protein expression in K562 cells. It has been reported that some chemotherapy agents enhance Mcl-1 proteasome degradation resulting in increased expression of the double-strand DNA break marker γH2AX, DNA damage and apoptosis [22, 23]. In the present study we show that CM363 reduces Mcl-1 and increases γH2AX after 48 h of treatment which indicates that K562 cells cannot overcome cell cycle arrest and they are destined for apoptosis. These results further support the hypothesis that CM363-induced Mcl-1 downregulation may play a crucial role in the mechanism by which CM363 induces apoptosis in K562 cells.

The resistance to TKI has produced that many researchers focus their efforts on finding new therapies or new compounds for use as adjuvants for conventional therapy. Targeting Stat5 has become an attractive approach to overcome TKI resistances [8–10]. Interestingly, in this study we provide evidence that CM363 acts synergistically with IM to inhibit Stat5 phosphorylation and cell viability in IM-sensitive K562 cells. Moreover, we observed that CM363 is equally effective against IM-sensitive and resistant K562 cells. These results open the possibility to use CM363 as coadjuvant therapy in CML. Finally, in addition to the *in vitro* results, when K562 cells were implanted in mice, CM363 (10 mg/kg; ip) potently suppressed the growth of tumor xenografts. Therefore, CM363 could be of interest in cancer therapy, and several observations [34, 36], including this study, argue for its potential use in leukemias.

In summary, the present study shows for the first time that CM363 is a multi-targeting agent which has potent antiCML effects *in vitro* and *in vivo*. Reduced survival of K562 cells after exposure to CM363 is associated with G2-M phase cell cycle arrest and apoptosis. CM363 can inhibit cell cycle progression, at least in part, by Chk2 activation, as well as decresing expression and/or activity of cyclin B and Cdc25. Induction of cell cycle arrest and apoptosis by CM363 were associated with inhibition of Bcr-Abl/Jak2/Stat5 signaling pathway and induction of JNK activity. Clinically relevant, the compound potentiates the antiproliferative activity of TKI inhibitor IM, circunvent IM-resistance in CML cells and suppressed the growth of human CML *in vivo*. Therefore, these data provide a rationale for the development of novel NPQ-based derivatives as therapeutic agents against human leukemia.

## MATERIALS AND METHODS

### Ethical statement

Investigation has been conducted in accordance with the ethical standards and according to the Declaration of Helsinki and according to the national and international guidelines and it has been approved by the authors’ institutional review board. All animal experiments were performed according to the protocol previously approved by the Bioethics Committee of the University of Salamanca, according to EU and national laws.

### Synthesis of CM363

CM363 [4 - (1, 6, 11 - trioxo - 2,3,4,6,11,12 – hexahydro - 1H - benzo [b] xanthen-12-yl) benzonitrile] (Figure 1A) was prepared according to EU/USA Patent Application PCT/EP2013/065552 (Fused quinonic compounds) [17].

### Reagents and antibodies

RPMI-1640, DMEM, DMEM-F12, McCoy’s 5A, fetal bovine serum (FBS), L-glutamine and PEST (50 units/ml penicillin, 50 μg/ml streptomycin) were provided by Lonza (Belgium). Recombinants human Erytropoyetin (EPO) and GH were kindly provided by Roche and Pfizer Spain laboratories, respectively. Interleukin-6 (IL6) and oncostatin M was provided by Miltenyi Biotec (Gladbach, Germany) and HumanZyme (Illinois, USA), respectively. The anti-CML drug IM [8] was purchased from Calbiochem (San Diego, USA). The origin of the different monoclonal and polyclonal antibodies used in the Western blotting analyzes were as follows: to pStat5, pBcr, Bcr, pJak2, Jak2, pSrc, Src, pERK1/2, ERK1/2, p38-MAPK, pJNK, pAKT, AKT, p27, cyclin D3, pRB^S780^, pRB^S807/811^, pchk1S296, pchk2T68, γH2AX, pS6, S6, cdc25C, Cyclin E, pStat3 and Stat3 were obtained from Cell Signaling Technology (Leiden, The Netherlands). Antibodies against β-actin, Stat5, JNK, cyclin B, Wee1, CDK2, Mcl-1, p21, BCL2, GAPDH and the horseradish peroxidase-conjugated secondary antibodies goat anti-rabbit and goat anti-mouse were obtained from Santa Cruz Biotechnologynology (CA, USA). Antibodies to caspase-3, -8 and -9 were obtained from Enzo Life Sciences (Lausen, Switzerland). Antibodies against cytochrome C, cyclin A, PARP, BUBR1 and BCL-X were obtained from BD Biosciences (Erembodegem, Belgium). Tri-Reagent and other generic chemicals were cited in this work were purchased from the Sigma-Aldrich, Roche Biochemicals (Mannheim, Germany), or Merck (Darmstadt, Germany). Enhanced chemiluminescent detection system was provided by Bio-Rad (München, Germany).

### Cells

All cell lines were purchased from the American Type Culture Collection (ATCC). The cell lines were growth at 37°C under 5% CO2 under humidified atmosphere. The cell lines K562 (derived from patients during the blast crisis phase of CML and IM sensitive), K562-R (IM resistance human CML) [31], KmycBT15I (KBTI; IM resistant human CML, expressing the Bcr-Abl T151I mutant) [32], HEL (human erythroleukemia), HL60 (human acute myeloid leukemia), MOLM-13 (human acute myeloid leukemia), MV4.11 (human acute monocytic leukemia), HCT-15 (human colorectal adenocarcinoma), PC3 (human prostate cancer), MCF7 (human breast cancer), NCTC3749 (mouse lymphoma) as well as the MRC.5 cells (non-malignant human lung fibroblasts) were maintained in RPMI-1640 medium. The cell lines L1210 (mouse lymphocytic leukemia cells), Raw (mouse macrophage), HeLa (human epithelial cervix cancer), U-2 OS (human osteosarcoma), MG63 (human osteosarcoma) and T47D (human breast cancer adenocarcinoma) were maintained in DMEM. HEK293 cells stably expressing the GH receptor (HEKGHR) were maintained in DMEM-F12 medium [51]. The human breast cancer cell line SKBR3 was maintained in McCoy’s 5A medium. Cell culture medium was supplemented with 10% FBS, L-glutamine (2 mM) and PEST. In addition, the cell culture medium for T47D was supplemented with 1 mM NaPyr and 10 mM HEPES. Human peripheral blood mononuclear cells (PBMC) were isolated from heparin-anticoagulated blood of healthy volunteers by centrifugation with Ficoll-Paque Plus (Amersham Biosciences).

### Cell viability assay

Cells were seeded in 96-well plates (BD Falcon, France) at exponential growth density (5000–20000 cells/well) and treated with vehicle (0.05% DMSO) or compound (0.01 to 10 μM) for 48–72 h. The mitochondrial metabolization of the tetrazolium salt 3-(4,5-methyltiazol-2yl-)-2,5diphenyl-tetrazolium bromide] (MTT) (Applichen, Germany) was used as indicator of cell viability [52]. Optical density was measured at 595 nm with the iMark Microplate Reader (BioRad, CA, USA).

### Time-lapse photomicroscopy

Kinetic measurements of cell proliferation and cytotoxicity were carried out by using the IncuCyte^TM^ HD real time imaging system (Essen BioScience, Hertfordshire, United Kingdom). After seeding in a polylysine-coated 96 well-plates [53], K562 cells were treated with different concentrations of compound. This IncuCyte™ microscope permits the acquisition of automated phase contrast images. Individual images are processed by an imbedded contrast-based confluence algorithm, which compute monolayer confluence for each image and at each time point. Multiple images are collected per well and averaged to provide a representative statistical measure of confluence.

### Luciferase reporter gene assay

The HEKGHR cell line was used to determine the effects of compounds on GH-regulated Stat5 transcriptional activity. HEKGHR cells were transfected with pSPI-GLE1-Luc (1 μg) overnight using Metafectene Pro^®^ (Biontex, Germany). The β-galactosidase expression plasmid (0.05 μg) (Thermo Scientific, Illinois, USA) was used for controlling transfection efficiency. The stable reporter cell line HeLa/Stat3-luc (Panomics, Fremont, USA), was used to determine the effects of compounds on oncostatin M-regulated Stat3 transcriptional activity [54]. Cells were seeded in a 6-well culture plate (Nunc, Denmark) and they were serum deprived (0.5% FBS) for 16 h. Then, cells were treated with vehicle (0.05% DMSO) or compound (0.1 to 10 μM) during 1 h before rhGH (50 nM) [51] or oncostatin M (50 ng/ml) were added for 7 h for induction of Stat5- or Stat3-dependent transcriptional activities, respectively. Cells were lysed in Passive Lysis Buffer and luciferase activity was determined by the Luciferase Assay System (Thermo Scientific, Illinois, USA). Luciferase activity was measured using the microplate reader Fluoroskan Ascent FL (Labsystems). Results are expressed as relative luciferase units (RLU) per mg of protein content and β-galactosidase activity and normalized to those obtained for vehicle-treated control cells.

### Cell cycle analysis and evaluation of apoptosis

Unsynchronized K562 cells were treated with vehicle (0.05% DMSO) or compounds in the presence FBS as indicated in figure legends. Then, cells were fixed in 70% ethanol and incubated with propidium iodide (PI) in the presence of RNAse. Nuclei to the left of the 2N peak containing hypodiploid DNA were considered as apoptotic. Fluorescent microscopy and flow cytometric analysis of PI-stained nuclei were performed to evaluate cell cycle and viability by using a FACSCalibur cytometer and CellQuest software (BD Biosciences, Erembodegen, Belgium) [55]. Apoptosis was also determined by translocation of phosphatidylserine to the cell surface using the annexin V-FITC apoptosis detection kit (BD Pharmingen, Erembodegen, Belgium) according to the manufacturer’s protocol.

### Stat-DNA binding activity analysis

Cells were treated with vehicle (0.05% DMSO) or compounds in the absence FBS as indicated in figure legends. Then, cells were rinsed with cold PBS-vanadate (1 mM) followed by harvesting cytosolic and nuclear proteins, in the presence of phosphatase and protease inhibitor cocktail, by using a Nuclear Extract kit (Active Motif Inc., CA, USA) according to the protocol supplied by the manufacturer. Stat5- or Stat3-specific DNA-binding activities in nuclear extracts were measured by using a TransAM STAT kit (Active Motif, CA, USA). Protein concentration was measured by the Bio-Rad DC Assay (Bio-Rad CA, USA). Absorbance was quantified with the iMark Microplate Reader (BioRad, CA, USA).

### Immunoblotting

Cells were treated with vehicle (0.05% DMSO) or compounds in the presence or absence FBS as indicated in figure legends. Then, cells were rinsed with cold PBS-vanadate (1 mM) followed by preparation of whole lysis extract with RIPA (Pierce, Illinois, USA). Equal amounts of each sample were separated by SDS-PAGE and transfered onto nitrocellulose membranes. After being blocked with 1% BSA-1% Blotto (anti-phospho-antibodies) (Santa Cruz Biotechnology, CA, USA) or 5% blotto (anti-total-antibodies) (Santa Cruz Biotechnology, CA, USA) (both diluted in Tris buffered saline with 0.05% Tween 20), membranes were immunoblotted overnight at 4°C with dilution (1:1000) in the same buffer of blocking of primary phosphoprotein or total protein antibodies. Antibody-specific labeling was revealed by incubation with a HRP-conjugated goat anti-mouse secondary antibody (Santa Cruz Biotechnology, CA, USA) or a HRP-conjugated goat anti-rabbit secondary antibody (Santa Cruz Biotech, CA, USA) (1:5000) and visualized with the Immu-Star^TM^ WesternC^TM^ kit (Bio-Rad Laboratories, CA, USA) using the ChemiDoc XRS system (Bio-Rad Laboratories, CA, USA), and the image analysis program Quantity one (Bio-Rad Laboratories, CA, USA). To control for differences in loading and transfer efficiency across membranes, membranes were incubated with a monoclonal mouse anti-beta actin antibody (Santa Cruz Biotechnology, CA, USA) [56]. The phosphoprotein array analysis of K562 cells was carried out by using the Profiler Human Phospho-Kinase Array Kit ARY003B (R&D Systems, Minneapolis, MN, USA) according to the manufacturer`s instructions.

### Gene expression analysis by real-time quantitative PCR (qPCR)

K562 cells (0.3 × 10^6^ cells/ml) were treated with vehicle (0.05% DMSO, final concentration) or compounds, in the presence of FBS, as indicated in figure legends. The mRNA expression levels of genes were measured using qPCR as previously described [57]. Briefly, total RNA was isolated by homogenization of cells with TriReagent (Sigma, St. Louis, MO) according to the protocol supplied by the manufacturer. RNA yields were measured by UV absorbance and the quality of total RNA was analyzed by using an Experium (BioRad). All samples were treated with RNAse-free DNase (Promega, Madison, WI) and 2 μg of total RNA was reverse transcribed by using an iScriptTM kit (BioRad, CA, USA). Two microliters of cDNA served as a template in a 20-μl qPCR reaction mix containing the primers and SYBR Green PCR Master mix (Diagenode, Belgium). Quantification of the gene expression was performed with an ABI PRISM7000 SD PCR System. A relative standard curve was constructed with serial dilutions (1:1, 1:2, 1:4, 1:8) using a pool of the cDNA generated from all groups used in the study. The amplification program consisted of 1 cycle of 95°C for 10 min, followed by 45 cycles of 95°C for 15 sec, annealing for 10 sec, and 72°C for 30 sec. The fluorescent intensity was measured at a specific acquisition temperature for each gene. A dissociation protocol was performed to assess the specificity of the primers and the uniformity of the PCR generated products. Data were extracted and amplification plots generated with ABI SDS software. All amplifications were performed in triplicate, and C_t_ scores were averaged for subsequent calculations of relative expression values. The level of individual mRNA measured by qPCR was normalized to the level of the housekeeping gene GAPDH by using the Pfaffl method [58]. Exon-specific primers (Table 2) were designed by the Primer 3 program [59].

**Table 2:** Gene names and primer sequences (5′- 3′) used for real-time PCR

Gene bank	Gene Product	Gene	Forward primer	Reverse primer
NM_002467.4	myelocytomatosis oncogene	Myc	CCAGCAGCGACTCTGAGG	CCAAGACGTTGTGTGTTC
NM_002648.3	serine/threonine kinase	Pim	GCTCGGTCTACTCAGGCATC	CATTAGGCAGCTCTCCCCAG
NM_001289746.1	glyceraldehyde-3P-dehydrogenase	GAPDH	CCATGGAGAAGGCTGGGG	CAAAGTTGTCATGGATGACC

### Drug combination analysis

The endpoint for these analyzes was derived from MTT assays incorporating a third set of replicates, where cells were exposed to constant-ratio combinations of the test drug CM363 with IM. K562 cells were exposed to doubling dilutions of the individual drugs over a wide range. The degree of inhibition of cell viability in these assays relative to unexposed controls was designed the effect, which ranged from 0 (no inhibition) to 1 (cell death). The effects of the drug treatments were plotted and analyzed to produce computed estimations of the relative responses to either drugs alone or in combination. In this way, it is possible to detect synergistic, additive or antagonistic effects. Triplicate independent experiments were set up for each K562 cells/drug combination. In each case the analysis generates plots of Combination Index (CI) *versus* effect. The combination indexes (CI) less than 1, equal to 1, and greater than 1 indicate synergism, additive, and antagonism between the drugs, respectively. The CI curves can be represented as the average CI values at effects of 0.20, 0.50, 0.70 and 0.9 (IC20, IC50, IC70 and IC90, respectively). The dose-effect curves of single or combined drug treatment were analyzed by the median-effect method of Chou and Talalay using the Calcusyn Software (Biosoft, Cambridge, UK) [30].

### Antileukemic activity of CM363 in xenograft mice tumors

Six weeks old female athymic mice (NOD-SCID), were purchased from Charles River River (Barcelona, Spain), and were housed in a specifically designed pathogen-free isolation facility as reported previously [33]. The mice were subcutaneously inoculated into the right flank with 2.5 × l0^6^ K562 cells in 100 μL of RPMI-1640 medium and 100 μL of Matrigel. When tumors became palpable, mice were randomized into three groups (*n* = 8 per group); the control group (receiving vehicle alone- DMSO: PEG-400: sterile water at 5:25:70 (w/v)), the group receiving CM363 10 mg/Kg or the group receiving the positive control IM 40 mg/kg. The vehicle and CM363 treatments were administered *via* intraperitoneal injection (i.p.) every day except Saturday and Sunday. While IM was administered orally using the same schedule. The study was conducted over a 25 days period. Caliper measurements of the tumor diameters were performed twice per week, and the tumor volume was estimated as the volume of an ellipse using the following formula: V = *4/3n* × (a/2) × (b/2)^2^, where *a* and *b* correspond to the longest and shortest diameter, respectively. Mice were sacrificed when their tumor diameters reached 2 cm, or when they became moribund. Differences in tumor volumes between control and treated group were evaluated using the Mann-Whitney test.

### Statistical analysis

The significance of differences between groups was tested by one-way ANOVA, followed by post hoc comparisons of group means according to the GraphPad Prism 5 program (GraphPad Software, San Diego, CA). Statistical significance was reported if *P* < 0.05 was achieved. For graphing purposes in the qPCR analysis, the relative expression levels were scaled so that the expression level of the vehicle-treated control group equalled one. The concentration required to reduce cell viability/proliferation by 50% (IC50) was determined graphically using the curve fitting algorithm of the GraphPad Prism 5. Values are means ± SEMs from three independent experiments, each performed in triplicate.

## References

[1] Ren R (2005). Mechanisms of BCR-ABL in the pathogenesis of chronic myelogenous leukaemia. Nat Rev Cancer.

[2] Hantschel O, Warsch W, Eckelhart E, Kaupe I, Grebien F, Wagner KU, Superti-Furga G, Sexl V (2012). BCR-ABL uncouples canonical JAK2-STAT5 signaling in chronic myeloid leukemia. Nat Chem Biol.

[3] Sinclair A, Latif AL, Holyoake TL (2013). Targeting survival pathways in chronic myeloid leukaemia stem cells. Br J Pharmacol.

[4] Markova B, Albers C, Breitenbuecher F, Melo JV, Brummendorf TH, Heidel F, Lipka D, Duyster J, Huber C, Fischer T (2010). Novel pathway in Bcr-Abl signal transduction involves Akt-independent, PLC-gamma1-driven activation of mTOR/p70S6-kinase pathway. Oncogene.

[5] Warsch W, Walz C, Sexl V (2013). JAK of all trades: JAK2-STAT5 as novel therapeutic targets in BCR-ABL1+ chronic myeloid leukemia. Blood.

[6] Gallipoli P, Cook A, Rhodes S, Hopcroft L, Wheadon H, Whetton AD, Jorgensen HG, Bhatia R, Holyoake TL (2014). JAK2/STAT5 inhibition by nilotinib with ruxolitinib contributes to the elimination of CML CD34+ cells *in vitro* and *in vivo*. Blood.

[7] Jacobberger JW, Sramkoski RM, Frisa PS, Ye PP, Gottlieb MA, Hedley DW, Shankey TV, Smith BL, Paniagua M, Goolsby CL (2003). Immunoreactivity of Stat5 phosphorylated on tyrosine as a cell-based measure of Bcr/Abl kinase activity. Cytometry A.

[8] Quintas-Cardama A, Kantarjian H, Cortes J (2009). Imatinib and beyond—exploring the full potential of targeted therapy for CML. Nat Rev Clin Oncol.

[9] Warsch W, Kollmann K, Eckelhart E, Fajmann S, Cerny-Reiterer S, Holbl A, Gleixner KV, Dworzak M, Mayerhofer M, Hoermann G, Herrmann H, Sillaber C, Egger G (2011). High STAT5 levels mediate imatinib resistance and indicate disease progression in chronic myeloid leukemia. Blood.

[10] Quintas-Cardama A, Kantarjian HM, Cortes JE (2009). Mechanisms of primary and secondary resistance to imatinib in chronic myeloid leukemia. Cancer Control.

[11] Dufies M, Jacquel A, Robert G, Cluzeau T, Puissant A, Fenouille N, Legros L, Raynaud S, Cassuto JP, Luciano F, Auberger P (2011). Mechanism of action of the multikinase inhibitor Foretinib. Cell Cycle.

[12] Barrio S, Gallardo M, Arenas A, Ayala R, Rapado I, Rueda D, Jimenez A, Albizua E, Burgaleta C, Gilsanz F, Martinez-Lopez J (2013). Inhibition of related JAK/STAT pathways with molecular targeted drugs shows strong synergy with ruxolitinib in chronic myeloproliferative neoplasm. Br J Haematol.

[13] Berger A, Sexl V, Valent P, Moriggl R (2014). Inhibition of STAT5: a therapeutic option in BCR-ABL1-driven leukemia. Oncotarget.

[14] Schafranek L, Nievergall E, Powell JA, Hiwase DK, Leclercq T, Hughes TP, White DL (2015). Sustained inhibition of STAT5, but not JAK2, is essential for TKI-induced cell death in chronic myeloid leukemia. Leukemia.

[15] Kumagai Y, Shinkai Y, Miura T, Cho AK (2012). The chemical biology of naphthoquinones and its environmental implications. Annu Rev Pharmacol Toxicol.

[16] Wellington KW (2015). Understanding cancer and the anticancer activities of naphthoquinones - a review. RSC Advances.

[17] McNaughton SG, Estévez-Braun A, Jiménez-Alonso S, Gutiérrez-Ravelo A, Fernández-Pérez L, Diaz-Chico BN (2014). Fused quinonic compounds. International (US-E) patent.

[18] Levitzki A (2012). Tyrosine kinase inhibitors: views of selectivity, sensitivity, and clinical performance. Annu Rev Pharmacol Toxicol.

[19] Kawabe T, Suganuma M, Ando T, Kimura M, Hori H, Okamoto T (2002). Cdc25C interacts with PCNA at G2/M transition. Oncogene.

[20] Dai Y, Grant S (2010). New insights into checkpoint kinase 1 in the DNA damage response signaling network. Clin Cancer Res.

[21] Danial NN, Korsmeyer SJ (2004). Cell death: critical control points. Cell.

[22] Aichberger KJ, Mayerhofer M, Krauth MT, Skvara H, Florian S, Sonneck K, Akgul C, Derdak S, Pickl WF, Wacheck V, Selzer E, Monia BP, Moriggl R (2005). Identification of mcl-1 as a BCR/ABL-dependent target in chronic myeloid leukemia (CML): evidence for cooperative antileukemic effects of imatinib and mcl-1 antisense oligonucleotides. Blood.

[23] Belmar J, Fesik SW (2015). Small molecule Mcl-1 inhibitors for the treatment of cancer. Pharmacol Ther.

[24] Liu RY, Fan C, Garcia R, Jove R, Zuckerman KS (1999). Constitutive activation of the JAK2/STAT5 signal transduction pathway correlates with growth factor independence of megakaryocytic leukemic cell lines. Blood.

[25] Shiu RP, Paterson JA (1984). Alteration of cell shape, adhesion, and lipid accumulation in human breast cancer cells (T-47D) by human prolactin and growth hormone. Cancer Res.

[26] Badache A, Hynes NE (2001). Interleukin 6 inhibits proliferation and, in cooperation with an epidermal growth factor receptor autocrine loop, increases migration of T47D breast cancer cells. Cancer Res.

[27] Raitano AB, Halpern JR, Hambuch TM, Sawyers CL (1995). The Bcr-Abl leukemia oncogene activates Jun kinase and requires Jun for transformation. Proc Natl Acad Sci U S A.

[28] Ly C, Arechiga AF, Melo JV, Walsh CM, Ong ST (2003). Bcr-Abl kinase modulates the translation regulators ribosomal protein S6 and 4E-BP1 in chronic myelogenous leukemia cells via the mammalian target of rapamycin. Cancer Res.

[29] Dai CL, Shi J, Chen Y, Iqbal K, Liu F, Gong CX (2013). Inhibition of protein synthesis alters protein degradation through activation of protein kinase B (AKT). J Biol Chem.

[30] Chou TC (2010). Drug combination studies and their synergy quantification using the Chou-Talalay method. Cancer Res.

[31] Aceves-Luquero CI, Agarwal A, Callejas-Valera JL, Arias-Gonzalez L, Esparis-Ogando A, del Peso Ovalle L, Bellon-Echeverria I, de la Cruz-Morcillo MA, Galan Moya EM, Moreno Gimeno I (2009). ERK2, but not ERK1, mediates acquired and “de novo” resistance to imatinib mesylate: implication for CML therapy. PLoS One.

[32] Albajar M, Gomez-Casares MT, Llorca J, Mauleon I, Vaque JP, Acosta JC, Bermudez A, Donato N, Delgado MD, Leon J (2011). MYC in chronic myeloid leukemia: induction of aberrant DNA synthesis and association with poor response to imatinib. Mol Cancer Res.

[33] Ocio EM, Maiso P, Chen X, Garayoa M, Alvarez-Fernandez S, San-Segundo L, Vilanova D, Lopez-Corral L, Montero JC, Hernandez-Iglesias T, de Alava E, Galmarini C, Avilés P (2009). Zalypsis: a novel marine-derived compound with potent antimyeloma activity that reveals high sensitivity of malignant plasma cells to DNA double-strand breaks. Blood.

[34] Mao X, Yu CR, Li WH, Li WX (2008). Induction of apoptosis by shikonin through a ROS/JNK-mediated process in Bcr/Abl-positive chronic myelogenous leukemia (CML) cells. Cell Res.

[35] Sun J, McKallip RJ (2011). Plumbagin treatment leads to apoptosis in human K562 leukemia cells through increased ROS and elevated TRAIL receptor expression. Leuk Res.

[36] Maia RC, Vasconcelos FC, de Sa Bacelar T, Salustiano EJ, da Silva LF, Pereira DL, Moellman-Coelho A, Netto CD, da Silva AJ, Rumjanek VM, Costa PR (2011). LQB-118, a pterocarpanquinone structurally related to lapachol [2-hydroxy-3-(3-methyl-2-butenyl)-1,4-naphthoquinone]: a novel class of agent with high apoptotic effect in chronic myeloid leukemia cells. Invest New Drugs.

[37] Pardanani A, Lasho T, Smith G, Burns CJ, Fantino E, Tefferi A (2009). CYT387, a selective JAK1/JAK2 inhibitor: *in vitro* assessment of kinase selectivity and preclinical studies using cell lines and primary cells from polycythemia vera patients. Leukemia.

[38] Xiao H, Bid HK, Jou D, Wu X, Yu W, Li C, Houghton PJ, Lin J (2015). A novel small molecular STAT3 inhibitor, LY5, inhibits cell viability, cell migration, and angiogenesis in medulloblastoma cells. J Biol Chem.

[39] Flanagan ME, Blumenkopf TA, Brissette WH, Brown MF, Casavant JM, Shang-Poa C, Doty JL, Elliott EA, Fisher MB, Hines M, Kent C, Kudlacz EM, Lillie BM (2010). Discovery of CP-690,550: a potent and selective Janus kinase (JAK) inhibitor for the treatment of autoimmune diseases and organ transplant rejection. J Med Chem.

[40] Lu K, Fang XS, Feng LL, Jiang YJ, Zhou XX, Liu X, Li PP, Chen N, Ding M, Wang N, Zhang J, Wanx X (2015). The STAT3 inhibitor WP1066 reverses the resistance of chronic lymphocytic leukemia cells to histone deacetylase inhibitors induced by interleukin-6. Cancer Lett.

[41] Samanta A, Perazzona B, Chakraborty S, Sun X, Modi H, Bhatia R, Priebe W, Arlinghaus R (2011). Janus kinase 2 regulates Bcr-Abl signaling in chronic myeloid leukemia. Leukemia.

[42] Wang W, Wang YQ, Meng T, Yi JM, Huan XJ, Ma LP, Tong LJ, Chen Y, Ding J, Shen JK, Miao ZH (2014). MCL-1 degradation mediated by JNK activation via MEKK1/TAK1-MKK4 contributes to anticancer activity of new tubulin inhibitor MT189. Mol Cancer Ther.

[43] Horita M, Andreu EJ, Benito A, Arbona C, Sanz C, Benet I, Prosper F, Fernandez-Luna JL (2000). Blockade of the Bcr-Abl kinase activity induces apoptosis of chronic myelogenous leukemia cells by suppressing signal transducer and activator of transcription 5-dependent expression of Bcl-xL. J Exp Med.

[44] Riedl SJ, Salvesen GS (2007). The apoptosome: signalling platform of cell death. Nat Rev Mol Cell Biol.

[45] Cross TG, Scheel-Toellner D, Henriquez NV, Deacon E, Salmon M, Lord JM (2000). Serine/threonine protein kinases and apoptosis. Exp Cell Res.

[46] Zarubin T, Han J (2005). Activation and signaling of the p38 MAP kinase pathway. Cell Res.

[47] Xiao RZ, He CM, Xiong MJ, Ruan XX, Wang LL, Chen Y, Lin DJ (2013). Inhibition of extracellular signal-regulated kinase activity by sorafenib increases sensitivity to DNR in K562 cells. Oncol Rep.

[48] Zu K, Hawthorn L, Ip C (2005). Up-regulation of c-Jun-NH2-kinase pathway contributes to the induction of mitochondria-mediated apoptosis by alpha-tocopheryl succinate in human prostate cancer cells. Mol Cancer Ther.

[49] Bartek J, Bartkova J, Lukas J (2007). DNA damage signalling guards against activated oncogenes and tumour progression. Oncogene.

[50] Brenner AK, Reikvam H, Lavecchia A, Bruserud O (2014). Therapeutic targeting the cell division cycle 25 (CDC25) phosphatases in human acute myeloid leukemia—the possibility to target several kinases through inhibition of the various CDC25 isoforms. Molecules.

[51] Maamra M, Finidori J, Von Laue S, Simon S, Justice S, Webster J, Dower S, Ross R (1999). Studies with a growth hormone antagonist and dual-fluorescent confocal microscopy demonstrate that the full-length human growth hormone receptor, but not the truncated isoform, is very rapidly internalized independent of Jak2-Stat5 signaling. J Biol Chem.

[52] Mosmann T (1983). Rapid colorimetric assay for cellular growth and survival: application to proliferation and cytotoxicity assays. J Immunol Methods.

[53] Rainaldi G, Calcabrini A, Santini MT (1998). Positively charged polymer polylysine-induced cell adhesion molecule redistribution in K562 cells. J Mater Sci Mater Med.

[54] Stephens JM, Lumpkin SJ, Fishman JB (1998). Activation of signal transducers and activators of transcription 1 and 3 by leukemia inhibitory factor, oncostatin-M, and interferon-gamma in adipocytes. J Biol Chem.

[55] Sanchez-Sanchez B, Gutierrez-Herrero S, Lopez-Ruano G, Prieto-Bermejo R, Romo-Gonzalez M, Llanillo M, Pandiella A, Guerrero C, Miguel JF, Sanchez-Guijo F, Cañizo Del, Hernández-Hernández A (2014). NADPH oxidases as therapeutic targets in chronic myelogenous leukemia. Clin Cancer Res.

[56] Guerra B, Olmedillas H, Guadalupe-Grau A, Ponce-Gonzalez JG, Morales-Alamo D, Fuentes T, Chapinal E, Fernandez-Perez L, De Pablos-Velasco P, Santana A, Calbet JA (2011). Is sprint exercise a leptin signaling mimetic in human skeletal muscle?. J Appl Physiol (1985).

[57] Fernandez-Perez L, Santana-Farre R, de Mirecki-Garrido M, Garcia I, Guerra B, Mateo-Diaz C, Iglesias-Gato D, Diaz-Chico JC, Flores-Morales A, Diaz M (2014). Lipid profiling and transcriptomic analysis reveals a functional interplay between estradiol and growth hormone in liver. PLoS One.

[58] Pfaffl MW (2001). A new mathematical model for relative quantification in real-time RT-PCR. Nucleic Acids Res.

[59] Rozen S, Skaletsky H (2000). Primer3 on the WWW for general users and for biologist programmers. Methods Mol Biol.

