# Correction: CM363, a novel naphthoquinone derivative which acts as multikinase modulator and overcomes imatinib resistance in chronic myelogenous leukemia

**DOI:** 10.18632/oncotarget.26008

**Published:** 2018-08-14

**Authors:** Borja Guerra, Patricia Martín-Rodríguez, Juan Carlos Díaz-Chico, Grant McNaughton-Smith, Sandra Jiménez-Alonso, Idaira Hueso-Falcón, Juan Carlos Montero, Rosa Blanco, Javier León, Germán Rodríguez-González, Ana Estévez-Braun, Atanasio Pandiella, Bonifacio Nicolás Díaz-Chico, Leandro Fernández-Pérez

**Affiliations:** ^1^ Instituto de Investigaciones Biomédicas y Sanitarias BioPharm Laboratory, Universidad de Las Palmas de Gran Canaria, Epaña; ^2^ Unidad de Apoyo a la Docencia en Enfermería-Fuerteventura, Universidad de Las Palmas de Gran Canaria, España; ^3^ Instituto Canario de Investigación sobre el Cáncer, España; ^4^ Centro Atlántico del Medicamento, España; ^5^ Centro de Investigación del Cáncer, CSIC, Universidad de Salamanca, España; ^6^ Instituto de Biomedicina y Biotecnología de Cantabria, CSIC-Universidad de Cantabria, España; ^7^ Departamento de Química Orgánica, Instituto de Bio-Orgánica Antonio González, Universidad de la Laguna, España

**This article has been corrected:** The correct author name is given below:

**Rosa Blanco**

Original article: Oncotarget. 2017; 8:29679-29698. https://doi.org/10.18632/oncotarget.11425

